# Two-Dimensional Topology Optimized Nonlocal Metasurfaces for Augmented Reality

**DOI:** 10.1021/acs.nanolett.5c05872

**Published:** 2026-03-02

**Authors:** Chih-Yao Hsu, Huan-Teng Su, Wan-Tzu Kuo, Wei-Zhe Li, Yu-Tzu Liu, Yu-Chuan Chang, Yao-Wei Huang

**Affiliations:** Department of Photonics, College of Electrical and Computer Engineering, 34914 National Yang Ming Chiao Tung University, Hsinchu 300093, Taiwan

**Keywords:** topology optimization, nonlocal metasurfaces, high quality factor, RGB channels, optical combiner

## Abstract

Metasurfaces have been widely explored in augmented reality (AR) platforms to replace bulky optical components. However, metasurface-based free-space combiners lack spectral multifunctionality capability, limiting the separation between ambient light and display light. While nonlocal metasurfaces offer a potential solution, existing designs typically rely on vertical stacking or spatial multiplexing yet often suffer from reduced efficiency. Here, we demonstrate a topology-optimized nonlocal metasurface that achieves high-*Q* resonances at RGB wavelengths via first-order reflective diffraction, enabling compact, multifunctional free-space combiners for AR. By introducing two-dimensional design freedom into resonant waveguide gratings, we realize freeform structures with high diffraction efficiencies, narrow spectral bandwidths, and precise wavelength selectivity. Experimental results validate close agreement with simulations, showing vivid color reproduction and a strong suppression of spectral leakage. Integrated into a free-space AR platform, our metasurface achieves high color purity with reduced display power, offering a promising path toward advanced optical displays and spectrally selective photonic systems.

## Graphical abstract

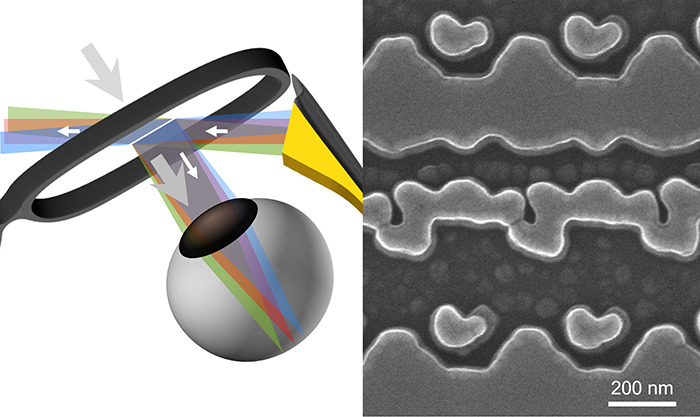

Metasurfaces combine the flexibility of diffractive optics with subwavelength control, enabling either broadband functionality or multiple narrowband responses in compact devices.^1,2^ Achromatic metalenses achieve broadband operation by compensating dispersion, with demonstrations across visible, infrared regimes, and others.^3–9^ Such designs typically rely on *local* responses, where each meta-atom acts independently. In contrast, *nonlocal* metasurfaces harness collective resonances, where optical behavior depends strongly on neighboring elements.^10–13^ Key platforms include quasibound states in the continuum (q-BICs),^14–19^ relying on symmetry breaking to couple trapped modes to free space, and resonant waveguide gratings (RWGs),^20–23^ also known as guided-mode resonance (GMR) gratings, that produce narrowband resonances via phase-matched leaky guided modes. Other nonlocal mechanisms or high-*Q* phenomena—such as Mie resonances,^24,25^ Fabry–Perot resonances,^26,27^ high-order multipoles,^28^ photonic crystal cavities,^29^ and structural defects^30^—offer various degrees of temporal (*Q*-factor) and spatial (diffractive) control over light.^31^

Nonlocal metasurfaces exhibit distinct spectral characteristics, depending on their design. Single-layer q-BIC metasurfaces typically support a narrowband resonance at one wavelength (Figure 1a),^32^ while multiple resonances can be achieved via spatial multiplexing or vertical stacking (Figure 1b).^14^ GMR-based metasurfaces naturally support multiple resonances across the spectrum (Figure 1c).^33^ In our prior work, we applied one-dimensional (1D) topology optimization to enhance their efficiency and color selectivity.^34^ However, spectral coupling among resonances remained a challenge, as the channels rose and fell together. Although lossy materials have been explored to suppress unwanted channels,^20^ independent spectral control remains difficult.

**Figure 1:**
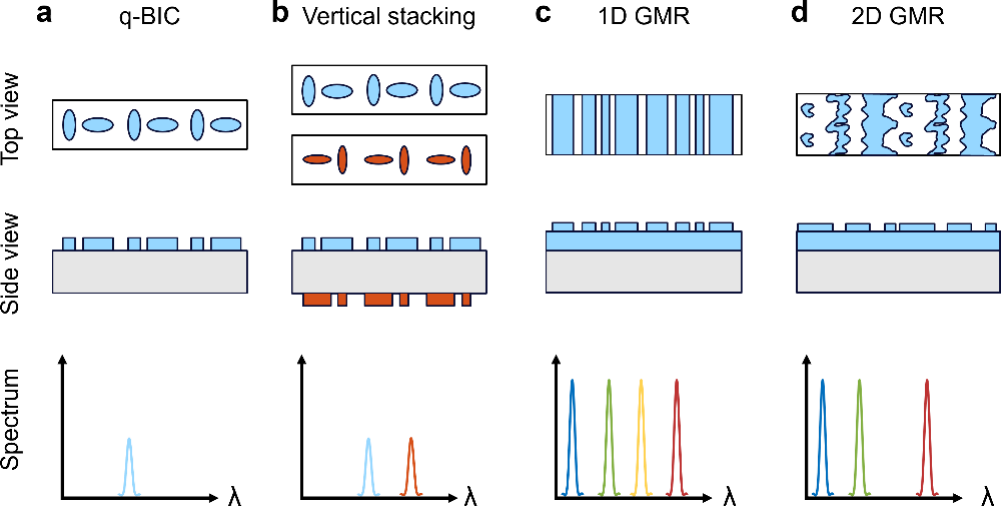
Comparison of nonlocal metasurface architectures. (a) A forward-designed single-layer q-BIC-based metasurface diffracts a single narrowband wavelength. (b) Multiple narrowband wavelengths can be diffracted using forward-designed metasurfaces through vertical stacking (or spatial multiplexing or interleaved) of structures. (c) An inverse-designed GMR-based metasurface using 1D topology optimization achieves multiple resonances with improved efficiency compared to forward designs. (d) A metasurface designed with 2D topology optimization enables simultaneous multiwavelength diffraction and suppression of undesired resonances by utilizing the additional design freedom in both lateral dimensions.

Here, we introduce a two-dimensional (2D) topology optimization framework that enables independent control over multiple GMR resonances. By expanding the design space to two dimensions within the grating layer, we achieve freeform structures (Figure 1d) that selectively retain red, green, and blue (RGB) channels while suppressing undesired modes. Various inverse design methods have been proposed for metasurface design,^35,36^ each with its strengths and trade-offs. Our method builds on the strengths of topology optimization,^34,37^ allowing freeform yet periodic designs within a manageable computational budget. By tailoring the optimization objectives, we realize high-*Q*, high-efficiency metasurfaces with strong spectral selectivity.

To validate a practical use case, we implemented our metasurface as an optical combiner in a free-space AR platform. This configuration allows simultaneous viewing of real and virtual scenes with enhanced brightness and reduced front projection. Recent advances in AR have explored metasurfaces to replace bulky optical components, including RGB-achromatic metalenses,^38,39^ display technologies,^40–42^ waveguide-type combiners,^43–46^ and free-space combiners.^14,47–49^ Compared with previous metasurface-based free-space combiners, our single-layer design offers sharper spectral control and simpler integration. Overall, this study demonstrates how 2D topology optimization expands metasurface functionality, supporting RGB channel separation and enabling compact, low-leakage AR systems.

Our metasurfaces are built upon an RWG structure, composed of a 160 nm-thick TiO_2_ grating layer deposited atop a 412 nm-thick TiO_2_ waveguide layer on a glass substrate. The GMRs are primarily governed by the waveguide thickness. Once this thickness is fixed, the primary operating wavelengths are effectively determined. Therefore, our optimization focuses on enhancing the diffraction efficiency by tailoring the freeform geometry of the grating layer. We select transverse electric (TE) mode with a grating period in *x*-direction (Λ_*x*_) of 870 nm yield theoretical resonances near 470.3 nm (blue), 532.0 nm (green), 597.6 nm (yellow), and 651.4 nm (red), with corresponding diffraction angles ranging from 30° to 50°. Building on our previous work,^34^ we extended the optimization to a 2D approach, providing additional design freedom in both lateral dimensions. In practice, the grating period in the *y*-direction (Λ_*y*_) is selected as 266 and 399 nm among our different samples. This constraint ensures that no first-order diffraction arises in the *y*-direction within the visible spectrum while still allowing in-plane momentum engineering. Detail initial design parameters are discussed in Supporting Information S1.

The topology optimization begins with a random distribution representing the initial permittivity profile at the grating layer, with permittivity spanning from that of air to that of TiO_2_. To ensure fabrication feasibility, a blur function is applied to refine this random pattern, while a contrast function drives the material pattern toward either air or TiO_2_. To guide this iterative process, we compute a figure of merit (FOM) and its gradient (∇FOM) at each iteration. The FOM is defined as

1 $$\mathrm{FOM}=\overset{N}{\underset{i=1}{\sum }}{\left|r_{\lambda i}\right|}^{2}$$

where ${\left|r_{\lambda i}\right|}^{2}$ is the first-order reflective diffraction efficiency at each target wavelength λ_*i*_. This summation can be tailored to emphasize different operating wavelengths, depending on whether single- or multiwavelength performance is desired. For rapid evaluations, we adopt an open-source rigorous coupled-wave analysis (RCWA) solver to calculate the FOM.^50^ To obtain the gradient of FOM efficiently, we utilized the adjoint method, which requires only one forward and one adjoint calculation per iteration. After each gradient-based update, we checked whether one of the stopping criteria is reached: (1) a maximum of 150 optimization iterations or (2) convergence to a local optimum beyond which further improvements are negligible. Upon meeting a stopping criterion, the final grating pattern undergoes a final sequence of postprocessing steps. The detailed procedure is discussed in Supporting Information S2.

To demonstrate the color selectivity of our 2D metasurface, we first optimized a grating targeting a single wavelength of 532.0 nm (G channel), termed Sample G. The *y*-direction periodicity was set to 266 nm, and the grating pattern was constrained to be laterally symmetric. After topology optimization, the resulting structure (right column of Figure 2b) features 3 TiO_2_ subregions (in blue) within each period. Simulation of the diffraction spectrum (left column of Figure 2b) reveals an extremely narrow bandwidth and high efficiency centered at the green wavelength. Under normal incidence, the first-order reflective diffraction angle follows the grating equation ${\theta_{diff}=\sin}^{-1}\left({\lambda /\Lambda }_{x}\right)$ , where λ denotes the wavelength. Detailed performance metrics, including peak wavelength, diffraction efficiency, diffraction angle, and *Q*-factor, are summarized in Table 1. Details of the *Q*-factor acquirements can be found in Supporting Information S3. Notably, the diffraction efficiencies for the red, yellow, and blue guided-mode wavelengths of Sample G are all less than half that of green, confirming the enhanced spectral selectivity achievable through 2D grating optimization. This result exemplifies the nonlocal nature of the metasurface and highlights the efficiency benefits afforded by topology optimization.

**Figure 2:**
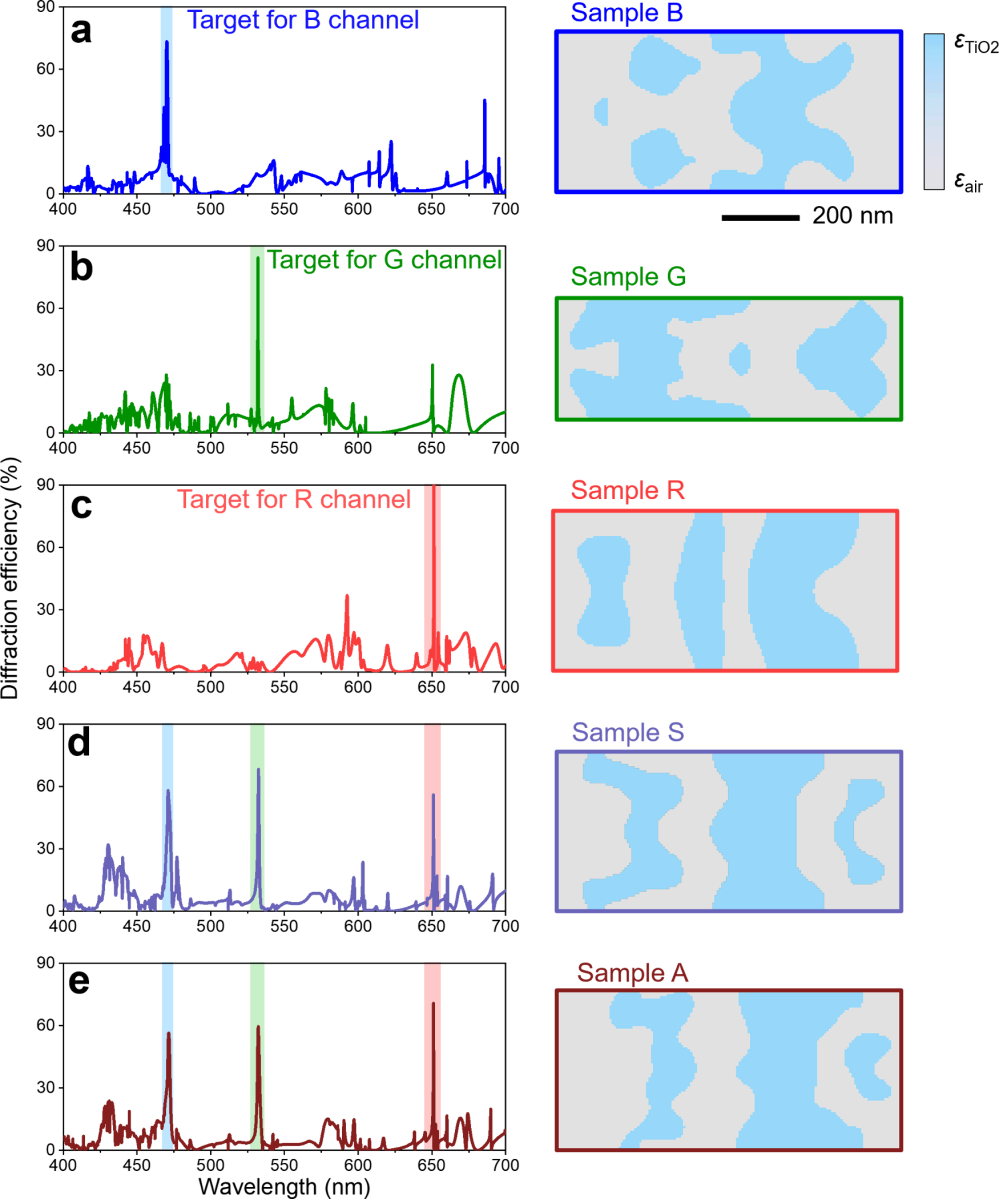
Optimized grating designs and their corresponding diffraction spectra. (a-c) Results of single-channel optimizations. Right column: Optimized freeform grating patterns for the blue (a, Sample B), green (b, Sample G), and red (c, Sample R) channels, respectively. Left column: The corresponding first-order diffraction efficiency spectrum for each case. (d,e) Results of multichannel (RGB) optimizations. Right column: Optimized freeform grating patterns under symmetric (d, Sample S) and asymmetric (e, Sample A) design constraints. The scale bar and colormap are shared among all grating pattern images and are indicated next to Sample B. Left column: The corresponding first-order diffraction efficiency spectra for each case.

**Table 1:** Numerical Specifications of Single-Wavelength Metasurfaces

	Sample B	Sample G	Sample R
Peak wavelength (nm)	470.3	532.0	651.4
Efficiency	73.4%	84.4%	90.9%
Diffraction angle (°)	32.7	37.7	48.5
*Q*-factor	304	2089	3596

To further highlight wavelength selectivity of our approach, we optimized two additional single-wavelength metasurfaces targeting 470.3 nm (Sample B) and 651.4 nm (Sample R). For these cases, the *y*-direction periodicity was increased to 399 nm to expand the optimization domain and permit more structural flexibility. The resulting freeform grating patterns and their simulated spectra are shown in Figures 2a and 2c, each exhibiting a dominant and narrow-band peak at the respective target wavelength. As summarized in Table 1, these metasurfaces exhibit narrow bandwidths and high diffraction efficiencies of up to 91%, thereby meeting the specified FOM criteria.

Compared to 1D-optimized designs,^34^ these 2D-optimized structures exhibit superior diffraction efficiencies and higher *Q*-factors, up to 3596 in simulation (Table 1). The *Q*-factor is influenced by the interplay between the GMR of the waveguide and the resonance behavior of the grating. Notably, the Mie-type resonances enabled by 2D freeform gratings tend to produce higher *Q*-factors than the Fabry-Pérot-type resonances typical of 1D gratings. Among the samples, Sample R achieves the highest *Q*-factor, owing to its operation near the fundamental GMR mode of the waveguide.

Building on the previous single-wavelength results, we next targeted our FOM at the 3 primary colors simultaneously, which together enable a broad range of color reproduction for display applications. Our objective was to simultaneously maximize diffraction efficiency at these 3 wavelengths while suppressing the yellow resonance observed in the 1D metasurface. For this multiwavelength design, the *y*-direction periodicity remained at 399 nm, and we considered both symmetric and asymmetric constraints for the grating pattern. Notably, the *y*-direction periodicity was intentionally chosen to lie between the free-space wavelength and the effective wavelength inside the TiO_2_ waveguide, such that additional degrees of freedom introduced by asymmetric designs remain confined to guided modes and do not result in far-field diffraction along the *y*-direction (see Supporting Information S1 for details). Figures 2d and 2e depict the simulated spectra for the symmetric (Sample S) and asymmetric (Sample A) designs, with their performance metrics summarized in Table 2. The resonance peaks match the target wavelengths, confirming the high-*Q* performance of our 2D topology-optimized metasurfaces. Moreover, the yellow resonance, previously observed in the 1D design, was effectively suppressed,^34^ demonstrating the ability of 2D optimization to independently control membrane modes without being constrained by the waveguide layer.

**Table 2:** Numerical Specifications of Multiwavelength Metasurfaces

	Sample S			Sample A		
Peak wavelength (nm)	471.0	532.4	651.0	471.5	532.2	651.1
Efficiency	58.2%	68.4%	56.2%	56.5%	59.7%	70.8%
Diffraction angle (°)	32.8	37.7	48.4	32.8	37.7	48.4
*Q*-factor	176	575	3576	264	323	3740

Removing the symmetric constraint led to further improvement: the asymmetric design significantly reduced side resonances (e.g., a secondary blue-region peak at ∼477.0 nm, originally 26.2% in Figure 2d) while maintaining near-optimal performance at the target RGB wavelengths. Although the peak wavelengths, efficiencies, and *Q*-factors exhibited minor variations, the side resonance at 477.0 nm was reduced to only 10% in the asymmetric design, indicating more favorable spectral interference and improved selectivity. It is worth noting that while our chosen asymmetric design offers advantages, not all asymmetric configurations will yield similar benefits. Moreover, adopting an asymmetric design may introduce additional fabrication complexity. These trade-offs must therefore be considered carefully evaluated based on specific project requirements. These results highlight that symmetry constraints serve as a controllable design parameter, mediating trade-offs between optimization complexity, accessible modal space, and spectral selectivity, rather than acting as a strict design requirement.

We further compared the band structures of the 1D design and the two 2D metasurfaces (Samples G and A). Both 2D designs exhibit a resonance landscape richer than that of the 1D counterpart, reflecting the increased lateral degrees of freedom. Moreover, varying the *y*-direction periodicity modifies the accessible in-plane momentum conditions, leading to distinct resonance distributions between Samples G and A. Momentum analysis and coupling-coefficient fitting indicate that the optimized operating wavelengths involve two quasidegenerate coupling channels with different efficiencies. Consequently, different target wavelengths selected during optimization can favor different combinations of coupling contributions. Detailed band-structure analysis is provided in Supporting Information S4.

We fabricated 1 mm-diameter metasurface samples using electron-beam lithography, atomic layer deposition, and high-density plasma reactive-ion etching, as detailed in Supporting Information S5. Scanning electron microscopy (SEM) images of Sample G and Sample A (Figures 3a and 3d) show that the fabricated TiO_2_ structure closely matches the designed patterns, with each unit cell forming a uniform array. First-order diffracted efficiency was measured using a custom-built angular-resolved spectrometer.^34^ The measured spectra of Sample G (Figures 3b and 3c) reveal a single narrow peak with a maximum efficiency of 31% and a *Q*-factor of 149. The central wavelength is red-shifted to 575 nm relative to the simulated value. As discussed in Supporting Information S6, this shift is attributed to a modified resonance landscape in the fabricated structure, where a Mie-type resonance becomes dominant due to thickness deviations and structural imperfections. Detailed experimental performance metrics are summarized in Table 3. For Sample A, the measured spectra (Figures 3e and 3f) demonstrate 3 distinct peaks corresponding to the designed 3 primary colors. The red peak, however, shows a more pronounced deviation from its target, likely caused by variations in the waveguide mode (details provided in Supporting Information S7).

**Figure 3:**
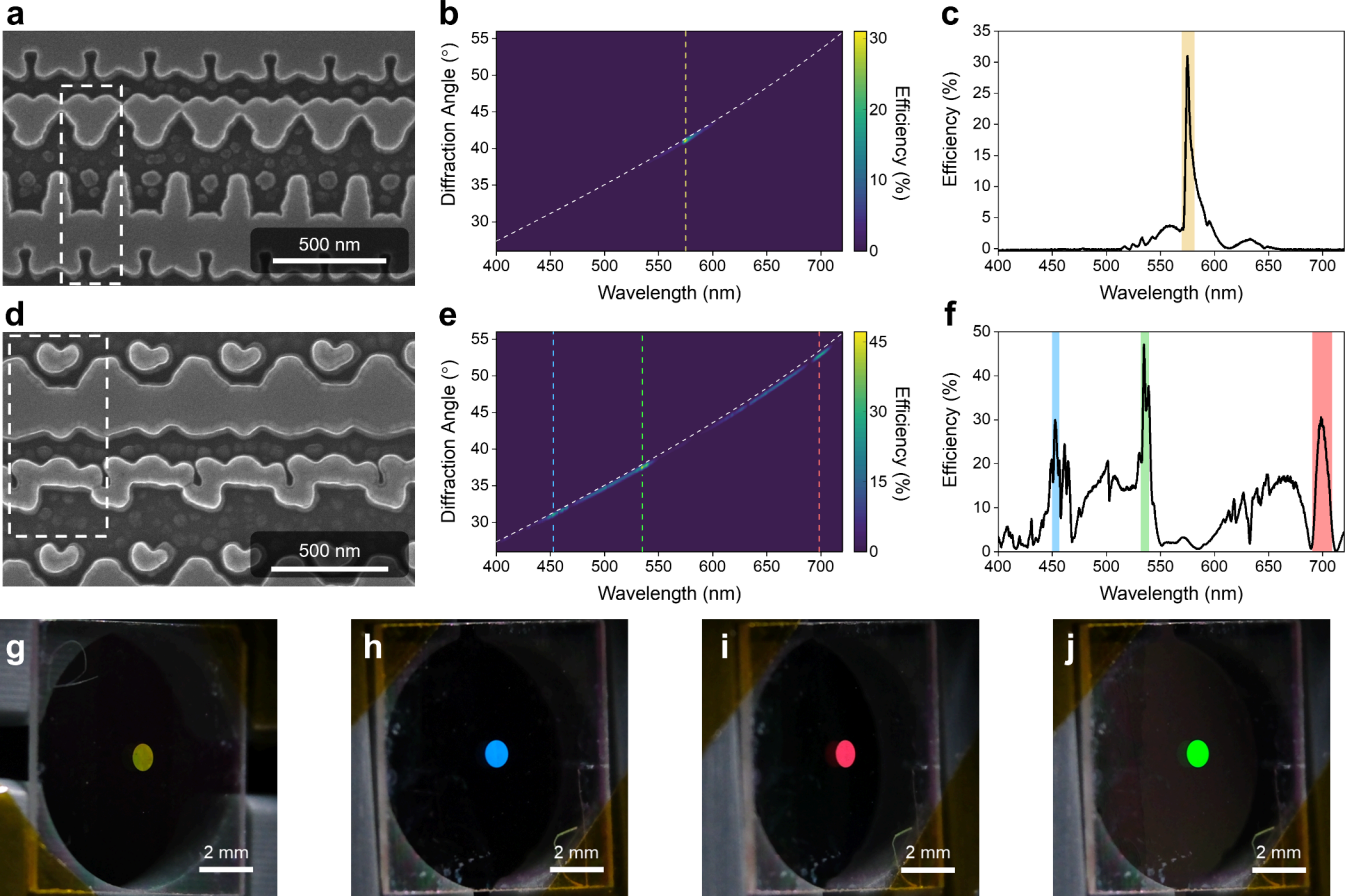
Experimental results for Sample G and Sample A. (a-c, g) Results for Sample G: SEM image (a), angular-resolved first-order diffraction spectrum (b), overall diffraction spectrum (c), and photograph captured at its operational angle (g). (d-f, h-j) Results for Sample A: SEM image (d), angular-resolved first-order diffraction spectrum (e), overall diffraction spectrum (f), and photographs captured at the operational angles corresponding to the RGB channels (h-j). White dashed rectangles in (a) and (d) indicate the unit-cell boundaries. Colored dashed lines in (b) and (e) and colored background bands in (c) and (f) denote the target operating wavelengths. White dashed lines in (b) and (e) indicate the theoretical diffraction angles.

**Table 3:** Experimental Specifications of Single and Multiwavelength Metasurfaces

	Sample G	Sample A			Sample A2		
Diameter (mm)	1	1			2		
Peak wavelength (nm)	575.0	451.6	534.7	698.5	468.2	526.3	663.9
Efficiency	31.0%	30.0%	47.1%	30.6%	41.6%	23.2%	24.8%
Diffraction angle (°)	41.4	31.3	37.9	53.4	32.6	37.2	49.7
*Q*-factor	149	148	95.3	69.8	153	104	105

To capture the vivid colors produced by the metasurface samples, we illustrate them with a white-light laser (spectrum provided in the Supporting Information S8) and photograph each sample at the angle corresponding to its peak diffraction efficiency using a camera equipped with a macro lens. Figure 3g shows a photograph of Sample G, where a bright monochromatic yellow appears exclusively at its diffraction angle, resulting from the red-shift of the designed green wavelength. By precisely tuning the detection angles, we obtained photographs of the Sample A at each of its 3 diffraction angles, as shown in Figures 3h–3j. The vivid colors demonstrate the metasurface’s sharp wavelength selectivity, highlighting its potential for applications requiring narrowband spectral control, such as anticounterfeiting or an optical combiner in an AR system.

Contemporary AR optical combiners, whether based on waveguide-type couplers or free-space architectures, often suffer from the low efficiency of virtual images and undesirable front projection. The former compromises the balance between virtual image brightness and real-world transparency, while the latter allows a portion of the display light to leak through the combiner, making it visible to bystanders. Figure 4a schematically illustrates the metasurface-based free-space combiner configuration and the corresponding propagation paths of ambient light and display light used to define these quantities. We define 2 physical quantities to evaluate the performance of the optical combiner: the display-to-ambient ratio (DAR) and the signal-to-leakage ratio (SLR). They are given as

2 $$DAR=\overset{®}{R_{1}\;}/\overset{®}{T_{env}\;}$$

3 $$SLR=R_{1}/T_{disp}$$

Here, *R*_1_ represents the first-order reflective diffraction spectrum, and $\overset{®}{R_{1}\;}$ denotes its average weighted by the display spectrum: $\overset{®}{R_{1}\;}=\int R_{1}\left(\lambda \right)S_{disp}\left(\lambda \right)d\lambda$ , where *S*_*disp*_(λ) is the spectral weighting function. This weighting accounts for the actual emission spectrum of the display source, ensuring that $\overset{®}{R_{1}\;}$ reflects the effective brightness perceived by the human eye rather than an unweighted spectral response. $\overset{®}{T_{env}\;}$ is the average ambient light transmittance over the visible spectrum (400–700 nm), measured from the substrate side of the combiner to the eye. And *T*_*disp*_ indicates the spectral forward leakage of display light, entering the structure from the display side and exiting toward bystanders. An ideal combiner should exhibit high DAR to ensure strong visibility of virtual content under ambient illumination and high SLR to suppress light leakage and reduce eye glow.

**Figure 4:**
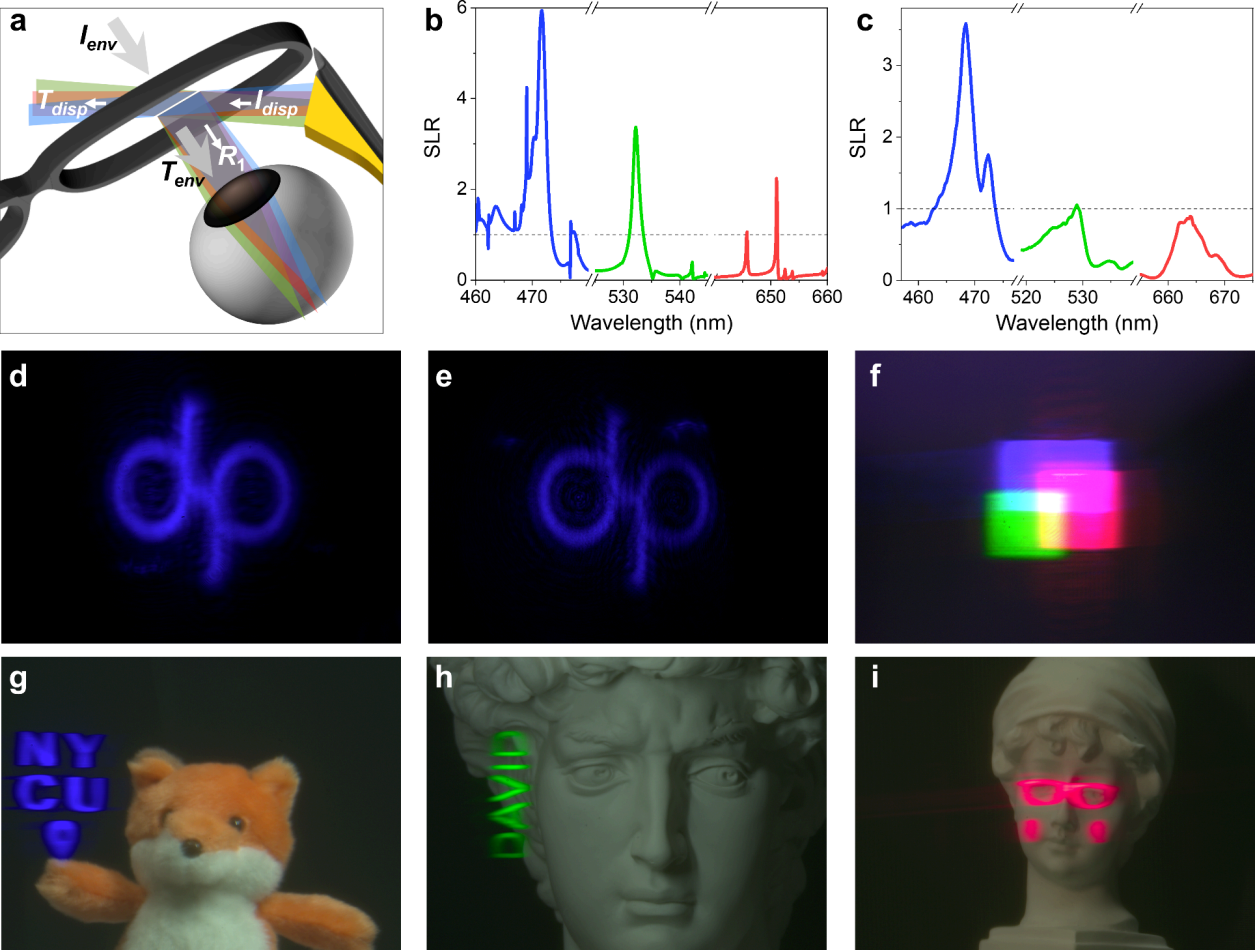
Demonstration of meta-optics-based free-space combiners in augmented reality. (a) Schematic illustration of the metasurface-based free-space combiner and the corresponding light paths associated with *R*_1_, *T*_*env*_, and *T*_*disp*_ in the DAR and SLR metrics. (b, c) Simulated (b) and experimental (c) spectra of the SLR. (d, e) AR images compare the virtual scene observed through the combiner in the *R*_1_ (d) and *T*_*disp*_ (e) configurations. (f–i) AR images under different illumination conditions: simultaneous RGB operation (f), blue-channel (g), green-channel (h), and red-channel (i).

To demonstrate the effectiveness of our high-*Q* metasurface (Sample A) as an optical combiner for AR, we implemented it in a free-space AR platform (Figure 4a). In our simulation, $\overset{®}{T_{env}\;}$ is about 34.8%, while $\overset{®}{R_{1}\;}$ from our metasurface (simulation) reaches around 32.5%, based on the spectral profile of our display system. This yields a DAR of 0.934 (detail provided in Supporting Information S9). As a practical example, consider a sunny outdoor environment with ambient luminance of approximately 5,000 nits. To achieve comparable virtual image brightness, the display only needs to emit about 5,353 nits of light (i.e., 5,000/0.934). This level of output is well within the capabilities of current commercial laser-based display systems.^51^ Moreover, by employing a narrower-bandwidth laser source, the DAR can be further increased, thereby reducing the required display brightness for the same visual impact. Additionally, the simulated SLR spectra are shown in Figure 4b (detail provided in Supporting Information S10), revealing strong spectral alignment and pronounced narrowband selectivity. Notably, the SLR reaches up to 5.94 in the blue channel, underscoring the metasurface’s inherent ability to suppress forward light leakage.

To expand the projection range of the virtual image, we fabricated a second metasurface, Sample A (2 mm in diameter, denoted as Sample A2). The experimental performance metrics are summarized in Table 3, indicating slight changes in peak wavelengths mainly because of fabrication error in waveguide thickness. In our experiment, $\overset{®}{T_{env}\;}$ was measured to be approximately 29.7%, while $\overset{®}{R_{1}\;}$ from our metasurface reached around 26.9%, based on the spectral profile of our display system. This yields an experimental DAR of 0.907 (Supporting Information S9), which is slightly lower but close to the simulated value. Additionally, the experimental SLR spectra are plotted in Figures 4c, exhibiting distinct peaks at the target wavelengths (detail provided in Supporting Information S10). To simplify the measurement process of *T*_*disp*_, we selected 3 representative incident angles, one for each spectral region corresponding to the target wavelengths. Although the experimental *Q*-factors and extinction ratios fall short of the simulated predictions, the SLR at the blue channel still achieves a 3.59, demonstrating that the design provides a tangible reduction of forward light leakage in practice.

Before performing the AR demonstration, we characterized the field of view (FOV) of Sample A2. A wavelength-tunable laser was used at wavelengths corresponding to the operating wavelengths of Sample A2. The collimated beam was expanded using beam expanders, and its polarization state was controlled with a linear polarizer. Owing to the small lateral size of the metasurface sample, an additional lens was employed to focus the beam onto the metasurface. The diffracted light was subsequently collected and imaged onto a camera through another lens. Detailed information regarding the optical components and their focal lengths is provided in Supporting Information S11. The overall optical configuration is analogous to a 4f system, enabling direct visualization of the band structure of the metasurface on the camera. The measured horizontal FOV is approximately 14°–17°, primarily limited by the focal lengths of the lenses used in the setup (see Supporting Information S11). We note that due to the presence of resonance bands and band gaps in the band structure, the captured diffraction images are not spatially uniform. Consequently, during the AR demonstration, the virtual image must be positioned within the brighter resonance region, which further reduces the effective usable FOV.

In our AR demonstration, virtual images were generated by spatially modulating a wavelength-tunable laser beam through a back-illuminated micro-LCD. Compared to the FOV characterization setup, the illumination optics were modified to enable image projection rather than angular band-structure mapping. Specifically, the laser beam was expanded and subsequently passed through the micro-LCD, where the image pattern was encoded. The modulated beam was then collimated and directed onto the metasurface sample, where wavelength-selective diffraction generated the virtual image. The diffracted light was collected by an “eyeball model” consisting of an imaging lens and a visible-light camera, approximating the perspective of a human observer. Additionally, the metasurface is partially transparent, allowing the camera to simultaneously capture both the real-world environment and the superimposed virtual images. Detailed information about each component can be found in Supporting Information S12.

Figures 4d and 4e compare the logo pattern under the *R*_1_ and *T*_*disp*_ configurations for the blue channel (peak SLR in Figure 4c). Owing to the 3.59-fold enhancement, the reflective mode markedly suppresses forward light leakage, yielding a brighter image while minimizing illumination observable by external viewers. The measurement procedure and the corresponding results for the green and red operating wavelengths are provided in Supporting Information S12. Across the three channels, the measured horizontal FOV ranges from 5.2° (blue) to 6.3° (red), reflecting the wavelength-dependent angular dispersion characteristics of the metasurface. In addition, the metasurface’s transparency enables the camera to capture the real-world scene and the superimposed virtual content in a single frame (Figure 4f–4i). Figure 4g shows NYCU’s mascot fox overlaid with a virtual “NYCU” and location marker, illustrating how brand identity and location information can be seamlessly integrated into real-world scenes. Figure 4h illustrates a scene where a facial recognition system identifies an object as “David”; instantly, a green virtual “DAVID” label appears and seamlessly integrates into the real-world statue *David*. Figure 4i depicts a statue of *British Girl*, augmented and adorned with virtual accessories to highlight how museum exhibits might offer multiple themed experiences by fusing different artistic styles. Figure 4f further illustrates a full-color virtual scene reconstructed by using the metasurface. Since the commercial micro-LCD and wavelength-tunable laser employed here cannot address all 3 primary colors simultaneously, the image was acquired sequentially at each channel and digitally combined.

It is worth noting that, as the metasurface operates in first-order reflective diffraction, chromatic dispersion is inherent to the system. A comparison between the input pattern and the eye-box photographs reveals that the red rectangle is suppressed along the horizontal (diffraction) axis relative to its blue and green counterparts (see Supporting Information S13). Consequently, the field of view exhibits anisotropy, with slightly different angular extents along the horizontal and vertical directions. Such wavelength-dependent shifts in magnification and position can be eliminated through calibration and precompensation, ensuring that the projected image matches the intended geometry. In the present study, however, the correction of the 3 images was performed during post processing rather than at the display stage.

Due to the dispersive nature of the metasurface, large-angle ambient illumination may produce weak transmissive diffraction components that manifest as rainbow-like artifacts or a *rainbow effect*. Numerical analysis (Supporting Information S14) shows that over most of the visible spectrum the directly transmitted ambient light dominates over the angularly dispersed diffracted components reaching the observer, indicating that the rainbow effect is generally secondary to direct transmission. Potential mitigation strategies include incorporating transmissive diffraction suppression into the topology-optimization figure of merit at the device level as well as introducing angular filtering elements (e.g., microlouver structures) at the system level to reduce large-angle ambient illumination. These approaches provide practical pathways for minimizing rainbow artifacts in future implementations.

Despite the success of this demonstration, several practical considerations remain. During projection, the relatively low LCD resolution can produce a pixelated image, which can be mitigated by using higher-resolution displays, such as fiber scanning near-eye display,^38^ flat panel laser display,^40^ and laser beam scanning display.^51^ Additionally, the Gaussian beam profile leads to brighter intensity at the center, but this can be partially corrected by the diffuser or adjusting the LCD’s brightness distribution. The intrinsic properties of the diffractive element may also introduce a slight blur band along the *y*-axis of the virtual image, and the projection range varies by wavelength due to diffraction-angle constraints. Moreover, we have not yet demonstrated simultaneous RGB image projection, a limitation of the specific micro-LCD used. While these aspects warrant further investigation, they do not fundamentally limit the feasibility or significance of our approach.

In this work, we numerically and experimentally demonstrated a 2D topology optimization strategy for designing high-*Q* nonlocal metasurfaces capable of achieving precise wavelength selectivity. By expanding the grating design space to two dimensions, our approach effectively suppressed undesired resonances and significantly enhanced the diffraction efficiencies at the target wavelengths. We demonstrated both single- and multiwavelength metasurface designs covering primary RGB colors, which exhibited narrow bandwidths and strong diffraction efficiencies.

The proposed framework combines forward physical design with inverse optimization: forward design establishes the accessible resonance conditions through material and structural parameters, while inverse optimization refines the in-plane grating geometry to enhance the coupling efficiency and spectral selectivity. Although demonstrated here for visible-wavelength RGB operation, the same workflow may be adapted to other spectral targets with appropriate choices of materials and structural parameters.

We fabricated prototypes and validated their performance through spectral measurements and direct imaging, both of which closely matched the simulation results. Notably, we integrated the optimized metasurface into a free-space augmented reality system, demonstrating high color purity and the practical potential to achieve bright virtual images with reduced display power.

Compared with other state-of-the-art metasurface-based free-space combiners (Table 4), our approach provides a unique combination of nonlocal operation, a high *Q*-factor, RGB channel support, and transmittance-mode imaging. While some prior works demonstrated high simulated reflectance (refs 47, 48 ) or multicolor operation (ref 14), most are either limited to local metasurfaces (refs 47, 49 ), lack experimental validation (ref 14), or only support reflectance-mode operation (refs 47, 48 ). In contrast, our design is experimentally validated, supports RGB operation through a single-layer GMR metasurface, and enables simultaneous see-through and color-selective display in free-space configurations—marking the first experimental realization of its kind.

**Table 4:** Performance Comparison of Our Device with State-of-the-Art Metasurface-Based Free-Space Combiners

	Reference 47	Reference 49	Reference 48	Reference 14	Our approach
building block	MIM antenna	MIM antenna	1D grating	q-BIC, stacking and interleaved patterns	GMR, single-layer pattern
material	Ag-SiO_2_-Ag	Au-resist-Au	resist	TiO_2_	TiO_2_
local/nonlocal	local	local	nonlocal	nonlocal	nonlocal
quality factor	low-*Q*	low-*Q*	mid-*Q*	high-*Q*	high-*Q*
reflectance (for display)	>90% (sim.)	48% (sim.)	95% (sim.)/84% (exp.)	∼32% (sim.)	71% (sim.)/42% (exp.)
transmittance imaging	no	yes	no	no	yes
reflectance imaging	yes	yes	yes	no	yes
channel(s)	1, red	1, red	1, green	3, RGB (sim.)	3, RGB

While challenges such as micro-LCD resolution, beam shaping, and fabrication tolerances remain, they do not fundamentally constrain the viability of this approach. We anticipate that continued improvements in metasurface design flexibility and nanofabrication techniques will further enable advanced display technologies, optical security, and other applications requiring precise spectral control. These results chart a promising course toward next-generation AR wearables and adaptive photonic systems demanding ultranarrowband functionality.

## Supplementary Material



## Data Availability

All data generated or analyzed during this study are included in this published article.

## Notes

Additional details on the composition of initial design parameters, detailed topology optimization procedure, calculation and analysis of *Q*-factor, band structure and momentum analysis, fabrication process, resonance at green and red channels, spectrum of white-light laser, calculation and analysis of DAR and SLR based on spectral data, FOV measurement of the AR platform, experimental setup for AR platform, dispersive image compression, and simulated analysis of rainbow effect (PDF)
