# Supplementary material for: Two-Dimensional Topology Optimized Nonlocal Metasurfaces for Augmented Reality
Source: Nano Lett. 2026 Mar 2;26(12):4080–8. doi: 10.1021/acs.nanolett.5c05872 (PMC13047679; doi:10.1021/acs.nanolett.5c05872)
Supplement: Supplementary file 1 [file nl5c05872_si_001.pdf]

Supporting Information for

Two-Dimensional Topology Optimized Nonlocal Metasurfaces for Augmented Reality

*Chih-Yao Hsu, Huan-Teng Su, Wan-Tzu Kuo, Wei-Zhe Li, Yu-Tzu Liu, Yu-Chuan Chang, and Yao-Wei Huang**

Department of Photonics, College of Electrical and Computer Engineering, National Yang Ming Chiao Tung University, Hsinchu 300093, Taiwan

*E-mail: ywh@nycu.edu.tw

Contents

S1. Initial design parameters

S2. Detailed topology optimization procedure

S3. Calculation and analysis of Q-factor

S4. Band structure and momentum analysis

S5. Fabrication Process

S6. Resonance at green channel

S7. Resonance at red channel

S8. Spectrum of white-light laser

S9. Calculation and analysis of DAR based on spectral data

S10. Calculation and analysis of SLR based on spectral data

S11. FOV measurement of the AR platform

S12. Experimental setup for AR platform

S13. Dispersive image compression

S14. Simulated analysis of rainbow effect

S1. Initial design parameters

To achieve operation at the red, green, and blue (RGB) primary colors, we first analyzed the transverse electric (TE) mode properties of a TiO_2 slab waveguide. We found that a waveguide thickness of 412 nm and a grating period in x-direction (Λ_x) of 870 nm yield theoretical resonances near 470.3 nm (blue), 532.0 nm (green), 597.6 nm (yellow), and 651.4 nm (red), with corresponding diffraction angles ranging from 30° to 50°. We set the grating thickness to 160 nm, which results in an aspect ratio of approximately 3, assuming a minimum feature width of 50 nm. A thicker grating would effectively increase the effective thickness of the waveguide and alter its modal properties, while a thinner would weaken coupling efficiency.

To suppress first-order diffraction in the y-direction, the grating period in y-direction (Λ_y) must satisfy the condition:

$$\frac{\lambda}{n_w} < \Lambda_y < \lambda, \tag{S1}$$

where n_w is the refractive index of the waveguide and λ is the operating wavelength. This constraint ensures that no first-order diffraction arises in the y-direction within the visible spectrum, while still allowing in-plane momentum engineering. Although increasing Λ_y offers greater design flexibility, it also increases computational complexity, requiring a trade-off between performance and feasibility.

S2. Detailed topology optimization procedure

The topology optimization begins with a random distribution representing the initial permittivity profile $\rho_0(x, y)$ at the grating layer, as shown in the top-left image of Figure S1(a). The permittivity spans from that of air to that of TiO_2. A blur function is then applied to refine this random pattern, transitioning it into a more spatially continuous distribution. This blurring step provides design freedom around the edges of the pattern while eliminating small, isolated features that are challenging to fabricate. Subsequently, a contrast function is applied to produce a high-contrast distribution that forces the material within the grating layer to be either air or TiO_2. In each iteration, the combined blur-contrast step introduces new perturbations, allowing the design process to escape local maxima and explore a broader portion of the solution space. Essentially, the contrast function emphasizes the binary nature of the material system (air versus TiO_2), promoting more pronounced boundaries between the two.

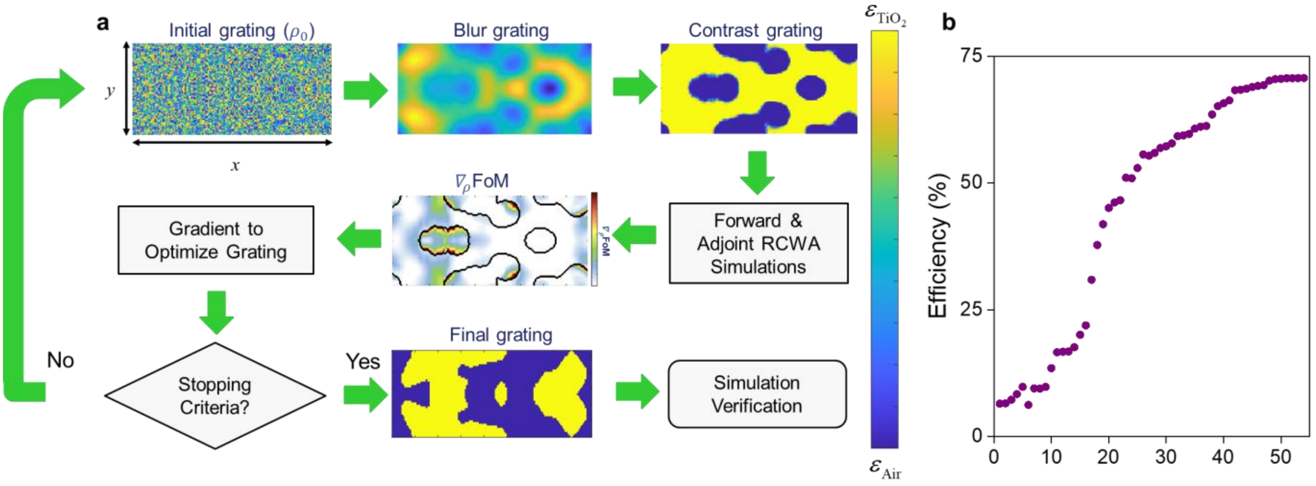

Figure S1. Process and evolution of topology optimization. (a) Flowchart illustrating the iterative optimization process. (b) Evolution of first-order diffraction efficiency, as a function of optimization iterations.

To guide this iterative process, we compute a figure of merit (FOM) and its gradient (∇FOM) at each iteration. The FOM is defined as

$$FOM = \sum_{i=1}^{N} |r_{\lambda i}|^2, \qquad \text{(S2)}$$

where $|r_{\lambda i}|^2$ is the first-order reflective diffraction efficiency at each targetwavelengths λ_i. This summation can be tailored to emphasize different operating wavelengths, depending on whether single- or multi-wavelength performance is desired.

For rapid evaluations, we adopt an open-source rigorous coupled-wave analysis (RCWA) solver to calculate the FOM. To obtain the gradient of FOM efficiently, we utilize the adjoint

method, which requires only one forward and one adjoint calculation per iteration. The gradient is given by

$$\nabla_\rho FOM(x,y) = \sum_{i=1}^{N} C|r_{\lambda i}|\mathrm{Re}\big[E_f(x,y)\cdot E_a(x,y)\big], \tag{S3}$$

where C is a constant determined by the mesh size and wavelength, while $E_f(x,y)$ and $E_a(x,y)$ are the electric fields at the grating layer obtained from the forward and adjoint simulations, respectively. We iteratively updated freeform distribution $\rho_{q+1}(x,y)$ using $\nabla_\rho FOM$ and the chain rule, accounting for the transformations introduced by the blur and contrast functions, where q denotes the iteration index.

Applying this gradient to the freeform grating pattern iteratively improves the FOM by increasing diffraction efficiency at the target wavelengths. An example optimization path corresponding to Sample B is illustrated in Figure S1(b). A sharp increase in efficiency is observed within the first 20 iterations, indicating fast convergence toward a local optimum. After each gradient-based update, we check whether one of the stopping criteria is reached: (1) a maximum of 150 optimization iterations or (2) convergence to a local optimum beyond which further improvements are negligible. Upon meeting a stopping criterion, the final grating pattern undergoes a final sequence of post-processing steps. These include an additional blur pass, contrast enhancement, and a binarization process to ensure well-defined material boundaries. Finally, we perform a high-resolution RCWA verification using higher harmonic orders to confirm that the optimized pattern delivers the desired optical performance.

S3. Calculation and analysis of *Q*-factor

The guided-mode resonances of the RWG-type nonlocal metasurface display the asymmetric line-shape characteristic of a Fano interference. Each cross section of the peak spectrum was modelled with the Fano profile

$$\sigma = A \times \frac{[q+\frac{2}{\Gamma_{res}}(E-E_0)]^2}{1+[\frac{2}{\Gamma_{res}}(E-E_0)]^2} + B, \tag{S4}$$

where A is the amplitude, q the Fano asymmetry parameter, E_0 the resonance center, Γ_{res} the FWHM, and B a constant background term. Nonlinear least-squares fitting with bounded parameters were applied to the corresponding resonances, yielding

$$Q = \frac{E_0}{\Gamma_{res}}. \tag{S5}$$

Small discrepancies between the experimental spectra and the fitted curves are mainly attributed to the overlap of multiple guided modes, which blurs the ideal Fano signature and thereby broadens the extracted linewidths.

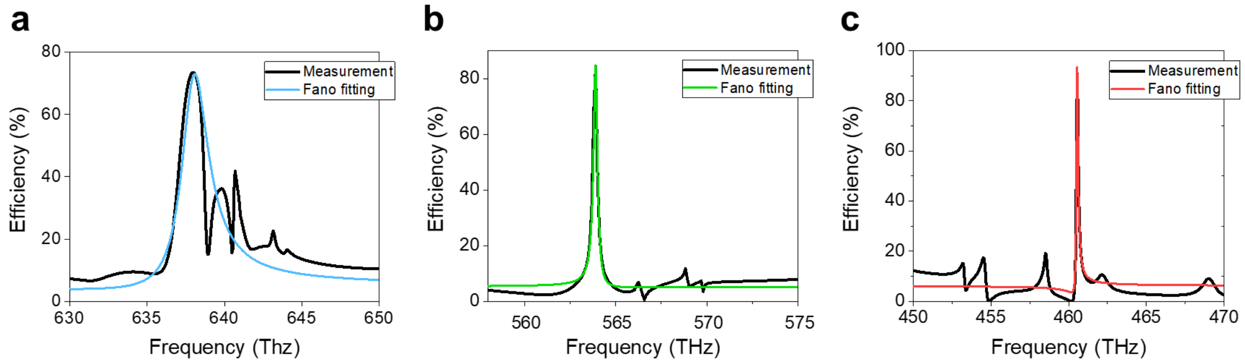

Figure S2. Simulated diffraction spectra of the metasurfaces with *Q*-factors with fitting of Fano resonance (attached to Table 1). **(a)** Sample B. **(b)** Sample G. **(c)** Sample R.

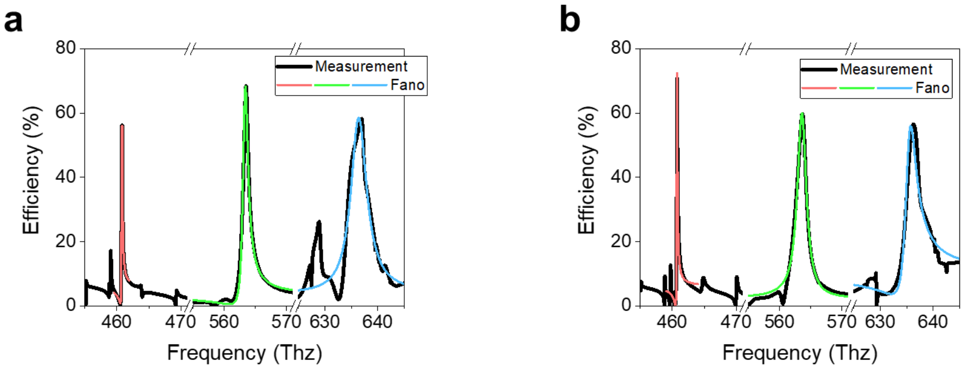

Figure S3. Simulated diffraction spectra of the metasurfaces with *Q*-factors with fitting of Fano resonance (attached to Table 2). **(a)** Sample S. **(b)** Sample A.

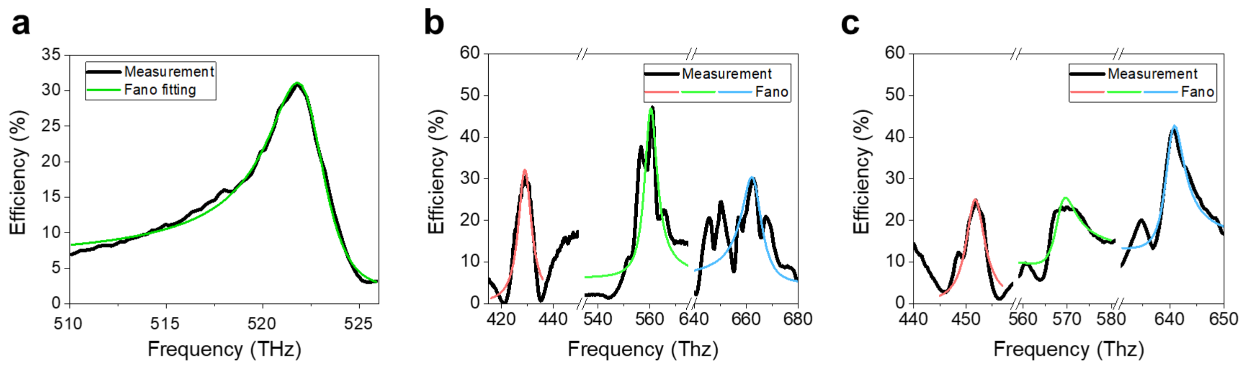

Figure S4. Experimental diffraction spectra of the metasurfaces with *Q*-factors with fitting of Fano resonance (attached to Table 3). **(a)** Sample G. **(b)** Sample A. (**c**) Sample A2.

S4. Band structure and momentum analysis

To analyze the underlying guided-mode resonances, we first consider a simplified planar slab waveguide model consisting of a $n_{air}/n_{TiO2}/n_{SiO2}$ three-layer structure, as illustrated in Figure S5(a). In this model, the grating layer is not explicitly included. Instead, the waveguide thickness is slightly adjusted to reproduce the resonance frequencies observed in full-wave simulations of the metasurface samples.

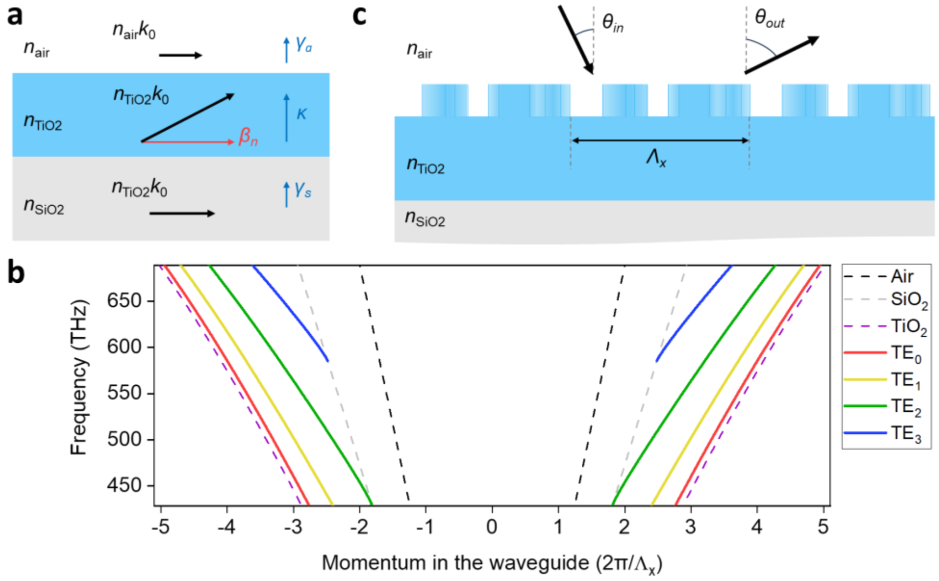

Figure S5. (a) Schematic of the simplified planar slab waveguide model consisting of air/TiO_2/SiO_2 layers. **(b)** Calculated band structure of the slab waveguide for TE modes. Dashed lines indicate the light lines of air, SiO_2 substrate, and TiO_2 waveguide, while solid curves correspond to discrete guided modes. (c) Schematic illustration of grating-assisted in- and out-coupling between free-space waves and guided modes, defining the momentum-matching conditions.

By applying electromagnetic boundary conditions, the eigenvalue equation for TE-polarized guided modes is obtained:

$$\tan(t\kappa) = \frac{\kappa(\gamma_a+\gamma_s)}{\kappa^2-\gamma_a\gamma_s}, \tag{S6}$$

where t denotes the waveguide thickness, κ is the transverse wavevector component inside the waveguide layer, and γ_a and γ_s represent the evanescent decay constants in the air and substrate regions, respectively. These quantities are related to the in-plane propagation constant β_n through

$$\kappa = \sqrt{(n_{TiO2}k_0)^2 - \beta_n^2}, \tag{S7}$$

$$\gamma_a = \sqrt{\beta_n^2 - (n_{air}k_0)^2}, \tag{S8}$$

$$\gamma_s = \sqrt{\beta_n^2 - (n_{SiO2}k_0)^2}, \tag{S9}$$

where $k_0 = 2\pi f/c$ is the free-space wavevector. Solving these relations yields the dispersion relation between frequency f and in-plane momentum β_n. Figure S5(b) shows the calculated band structure for $t = 424$ nm. The dashed lines indicate the light lines of air, glass substrate, and the waveguide layer, while the solid curves (red, yellow, green, and blue) correspond to the TE_0, TE_1, TE_2, and TE_3 modes, respectively.

The role of the grating is to enable coupling between free-space waves and guided modes while conserving in-plane momentum. The in-coupling and out-coupling processes satisfy:

$$k \sin\theta_{in} + m_{in}G = \beta_n, \tag{S10}$$

$$\beta_n + m_{out}G = k \sin\theta_{out}, \tag{S11}$$

where k is the incident wavevector, θ_{in} and θ_{out} denote the incident and diffraction angles (Figure S5(c)), $G = 2\pi/\Lambda_x$ is the grating momentum defined by the period Λ_x, and (m_{in}, m_{out}) are the grating coupling coefficients for in- and out-coupling, respectively. For first-order reflective diffraction, the relation $m_{in} + m_{out} = 1$ must be satisfied.

Figures S6(a–c) show the grating patterns of the 1D design, Sample G, and Sample A. The corresponding first-order reflective diffraction efficiency spectra, with incident momentum spanning from $-1G$ to approximately $+0.05G$, are shown in Figures S6(d–f). The calculated guided-mode band structure (Figure S5(b)) is folded into the same momentum interval to facilitate identification of the coupling conditions. From this analysis, the dominant coupling channels are identified as $(-3, +4)$ and $(+4, -3)$ for the 1D design, and $(-2, +3)$ and $(+3, -2)$ for Samples G and A.

The efficiency spectra of the 1D design (Figure S6(d)) exhibit several continuous linear features that closely follow the guided-mode dispersion relations, consistent with the relatively limited structural degrees of freedom. In contrast, Samples G and A display broader and more localized high-efficiency regions in the colormap, forming island-like features superimposed on the overall linear dispersion trends. This behavior reflects the increased design freedom of the 2D topology-optimized structures.

Regions of weaker efficiency indicate that the 2D designs support a greater number of resonances compared to the 1D configuration. This increase is primarily attributed to Mie-type scattering contributions arising from the 2D freeform grating geometry, which introduces additional localized resonant features beyond those predicted by the simple slab-waveguide model. In addition, some of these resonances are consistent with the presence of in-plane momentum components along the y-direction within the waveguide layer. For example, Sample G, which has a smaller y-direction periodicity than Sample A, exhibits fewer resonances in the

430–500 THz frequency range. This behavior is consistent with the reduced number of accessible momentum states associated with the smaller lateral period.

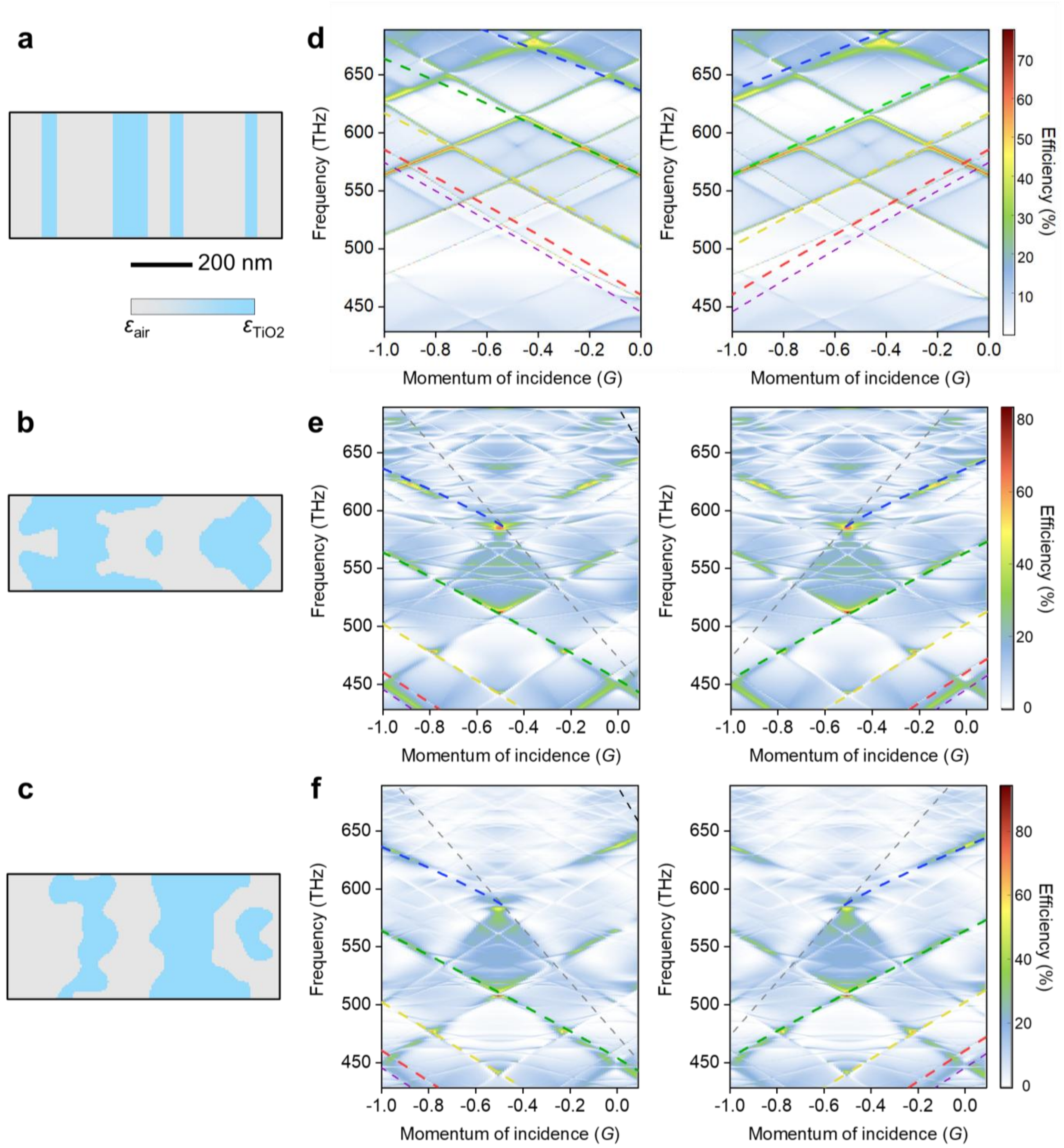

Figure S6. (a-c) Grating patterns of the 1D design (a), Sample G (b), and Sample A (c). (a), Sample G (b), and Sample A (c). **(d-f)** Colormaps of first-order reflective diffraction efficiency as a function of incident momentum for the corresponding structures. Dashed lines indicate the dominant coupling channels characterized by grating momentum coefficients (m_{in}, m_{out}) : (-3, +4) (left) and (+4, -3) (right) for the 1D design (d); (-2, +3) (left) and (+3, -2) (right) for Sample G (e) and Sample A (f).

Furthermore, several bands corresponding to $(-3, +4)$ and $(+3, -2)$ coupling channels exhibit quasi-degenerate behavior at the optimized operating wavelengths (470.3 nm, 532.0 nm, 597.6 nm, and 651.4 nm) under normal incidence. A positive m_{in} corresponds to forward propagation along the +*x* direction in the waveguide, which manifests as a positive slope in the band structure (positive group velocity), and vice versa. Although these quasi-degenerate bands occur at similar frequencies, they possess different coupling efficiencies. Therefore, optimization at different target wavelengths can preferentially select distinct combinations of coupling channels. For example, under normal incidence, the 1D design is more strongly associated with the $(-3, +4)$ coupling pathway, whereas Samples G and A more prominently exhibit the $(+3, -2)$ channel.

S5. Fabrication Process

To fabricate our metasurface, we began with a 1 × 1 cm^2 fused silica substrate onto which we deposited a 412-nm-thick TiO_2 waveguide layer using atomic layer deposition (ALD) with TDMAT and H2O precursors. Samples were then spin-coated with a 160-nm-thick electron beam resist (AR-P 6200.04, Allresist), followed by a thin conductive polymer layer (Espacer Z300, Showa Denko) to mitigate charging effects during electron beam lithography (EBL). An EBL system (Voyager, Raith) was then used to pattern the resist. After exposure, the conductive polymer layer was removed by water and dissolved regions were developed and removed by developer (AR 600-546, Allresist), leaving behind the inverse template of the desired grating structure. Subsequently, TiO_2 was deposited by using ALD. The ALD cycle continued until the patterned voids in the resist were fully filled. The excess TiO_2 on top of the resist was subsequently removed by high-density plasma reactive-ion etching (HDP-RIE), with the final etch depth confirmed via ellipsometry. Finally, the remaining resist and any residual nanostructures were removed by Remover PG, resulting in isolated, high-aspect-ratio TiO_2 freeform grating structure with minimal surface roughness.

S6. Resonance at green channel

For Sample G, the experimentally observed resonance is red-shifted to approximately 575 nm, compared to the simulated resonance near 532 nm. To investigate the origin of this discrepancy, we first incorporated structural information extracted from SEM images into the numerical model. Simulations were performed with scaled lateral dimensions, modified grating profiles, and adjusted waveguide and grating thicknesses. However, none of these variations produced a resonance shift sufficiently large to account for the experimentally observed wavelength.

We therefore analyzed the band structure of Sample G and compared it with the simplified planar slab-waveguide model. As shown in Figure S7(a), an additional resonance feature appears near 575 nm (highlighted by the red rectangle). This resonance exhibits an approximately horizontal dispersion in momentum space, indicating near frequency-independent behavior and correspondingly small group velocity. Such characteristics are consistent with a Mie-type resonance localized within the freeform grating geometry rather than a conventional guided-mode resonance.

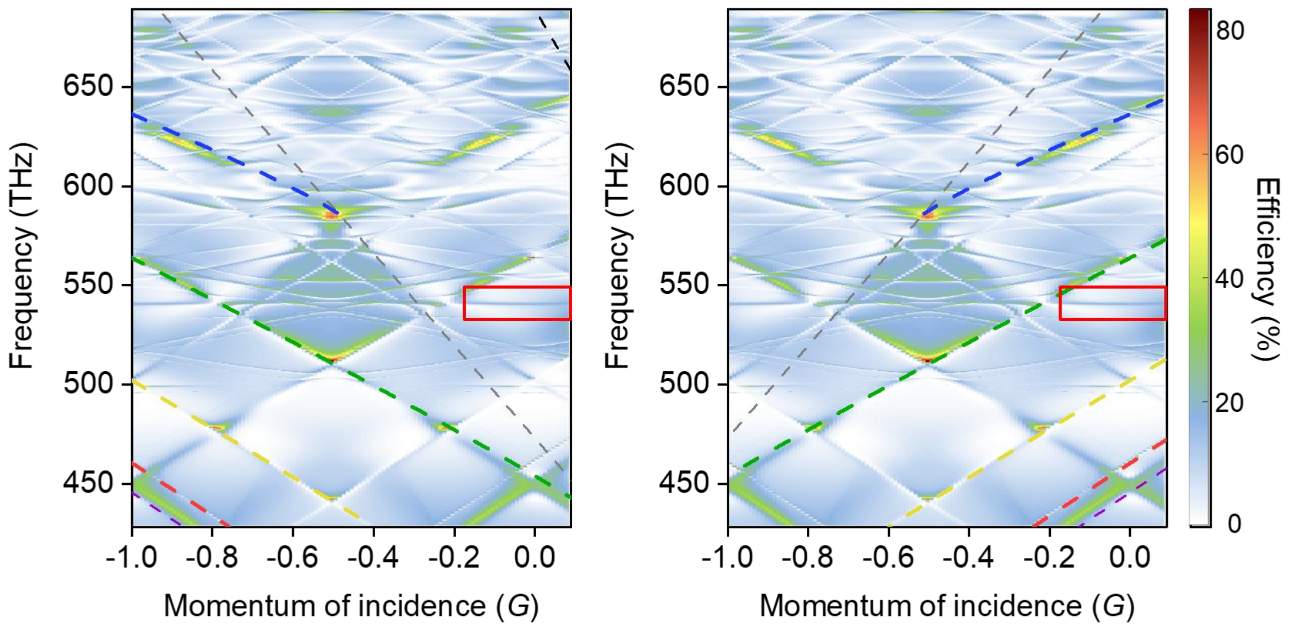

Figure S7. Colormap of first-order reflective diffraction efficiency as a function of incident momentum for Sample G. Dashed lines indicate the dominant coupling channels (m_{in}, m_{out}): (-2, +3) (left) and (+3, -2) (right) for Sample G. The red rectangle highlights a near frequency-independent resonance feature.

We propose that this Mie-type resonance becomes dominant in the fabricated structure due to deviations from the idealized geometry assumed in simulations. In particular, the numerical model assumes perfectly vertical and smooth grating sidewalls, whereas the

experimentally fabricated grating surfaces exhibit finite roughness and profile nonuniformity. These structural imperfections can enhance localized resonant behavior and modify the relative coupling strength between guided-mode and Mie-type resonances.

S7. Resonance at red channel

During fabrication, the TiO_2 waveguide layer was unintentionally over-deposited, resulting in a thickness larger than the intended 412 nm. To account for this deviation, we incorporated the increased thickness into the slab-waveguide momentum analysis.

Figure S8(a) shows the calculated band structure for a waveguide thickness of 474 nm. Compared with the nominal design, the thicker waveguide supports modified guided-mode dispersion and introduces additional resonance conditions. Under the grating-assisted momentum matching framework described in Supporting Information S4, the TE_2 mode satisfies the coupling condition at approximately 698 nm, where its in-plane momentum intersects the relevant grating momentum multiple. This intersection gives rise to a red-shifted first-order diffraction resonance.

To further verify the modal origin of this resonance, we simulated the electric field distribution under normal incidence at 698 nm. As shown in Figure S8(b), the RCWA-calculated E_y field profile exhibits the characteristic field distribution of the TE_2 mode within the waveguide layer. These results confirm that the experimentally observed red-shift arises from the thickness-induced modification of the guided-mode spectrum, which introduces an additional coupling channel relative to the nominal design.

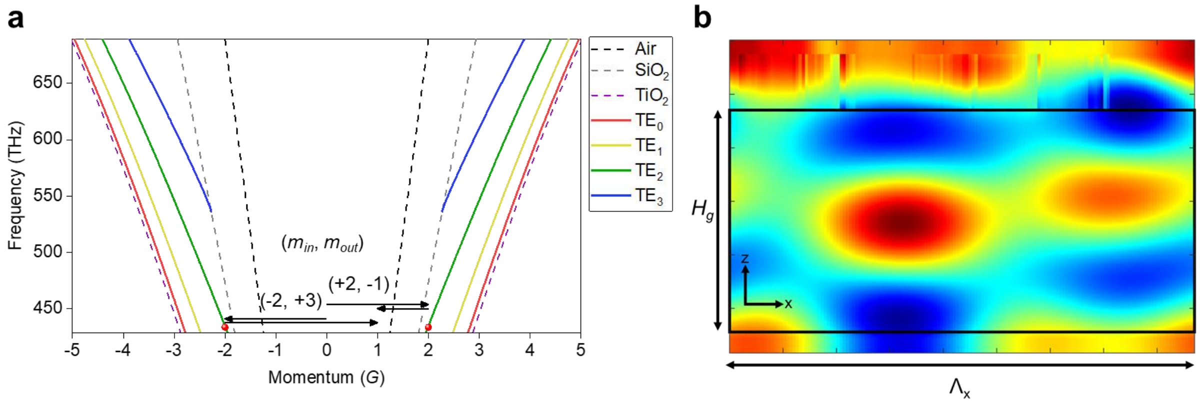

Figure S8. (a) Calculated band structure of the slab waveguide with a thickness of 474 nm. Dashed lines indicate the light lines of air, SiO_2 substrate, and TiO_2 waveguide, while solid curves correspond to discrete guided modes. Arrows mark the dominant grating-assisted coupling channels characterized by (m_{in}, m_{out}) = (-2, +3) and (+2, -1). **(b)** Simulated electric field distribution (E_y) inside the structure at 698 nm under normal incidence, confirming the TE_2 modal profile.

S8. Spectrum of white-light laser

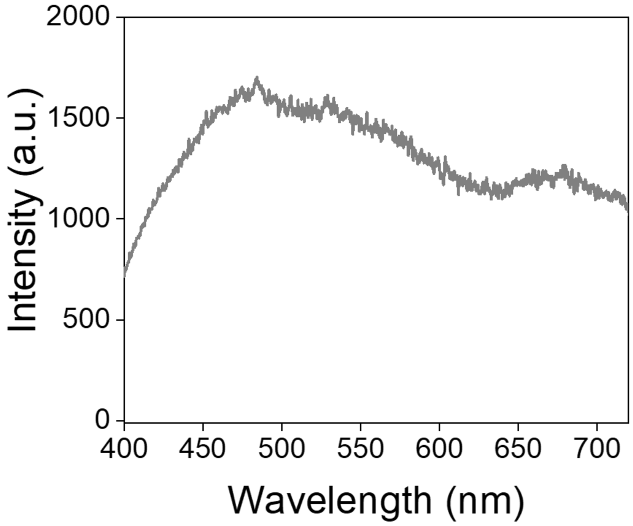

Figure S9. Spectrum of white-light laser for vivid color demonstration and spectrum measurement.

S9. Calculation and analysis of DAR based on spectral data

We compare the simulated and experimental spectra of T_{env} and R_1, along with the resulting DAR values. The simulated and measured spectra of T_{env} (Figures S10(a, d)) and R_1 (Figures S10(b, e)) are presented, while the spectral weighting functions $S_{dis}(\lambda)$, used to calculate the weighted average $\overline{R_1}$ are shown in Figures S10(c, f). Based on these results, DAR values of 0.934 (simulation) and 0.907 (experiment) are obtained.

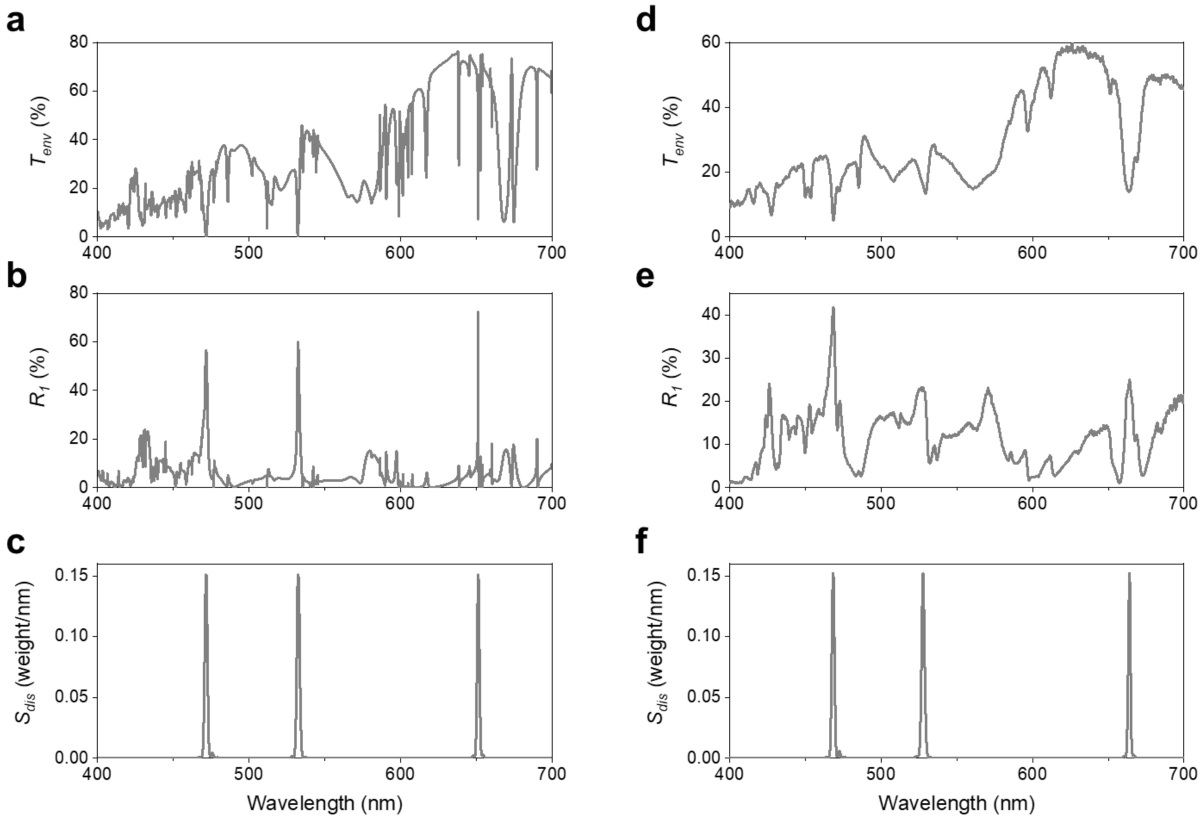

Figure S10. (a-c) Simulated results for Sample A. (a) Simulated ambient light transmittance T_{env}. (b) Simulated first-order reflective diffraction spectrum R_1. (c) Spectral weighting function $S_{dis}(\lambda)$, measured from our laser source operating at 471.5 nm, 532.2 nm, and 651.1 nm. Based on (a), the average ambient light transmittance $\overline{T_{env}}$ is calculated to be 34.8%. Combining (b) and (c), the weighted average diffraction efficiency $\overline{R_1}$ is 32.5%, resulting in a simulated DAR of 0.934. **(d-f)** Experimental results for Sample A2. (d) Measured ambient light transmittance T_{env}. (e) Measured first-order reflective diffraction spectrum R_1. (f) Spectral weighting function $S_{dis}(\lambda)$, measured from our laser source operating at 468.2 nm, 526.3 nm, and 663.9 nm. Based on (d), $\overline{T_{env}}$ is 29.7%, and from (e) and (f), $\overline{R_1}$ is 26.9%, resulting in an experimental DAR of 0.907.

S10. Calculation and analysis of SLR based on spectral data

We compare the simulated and experimental spectra of R_1 and T_{disp}, as well as the resulting SLR spectra. The simulated and measured spectra of R_1 (Figures 11(a, d)) and T_{disp} (Figures 11(b, e)) are presented. Based on these results, the SLR spectra (Figures 11(c, f)) are obtained.

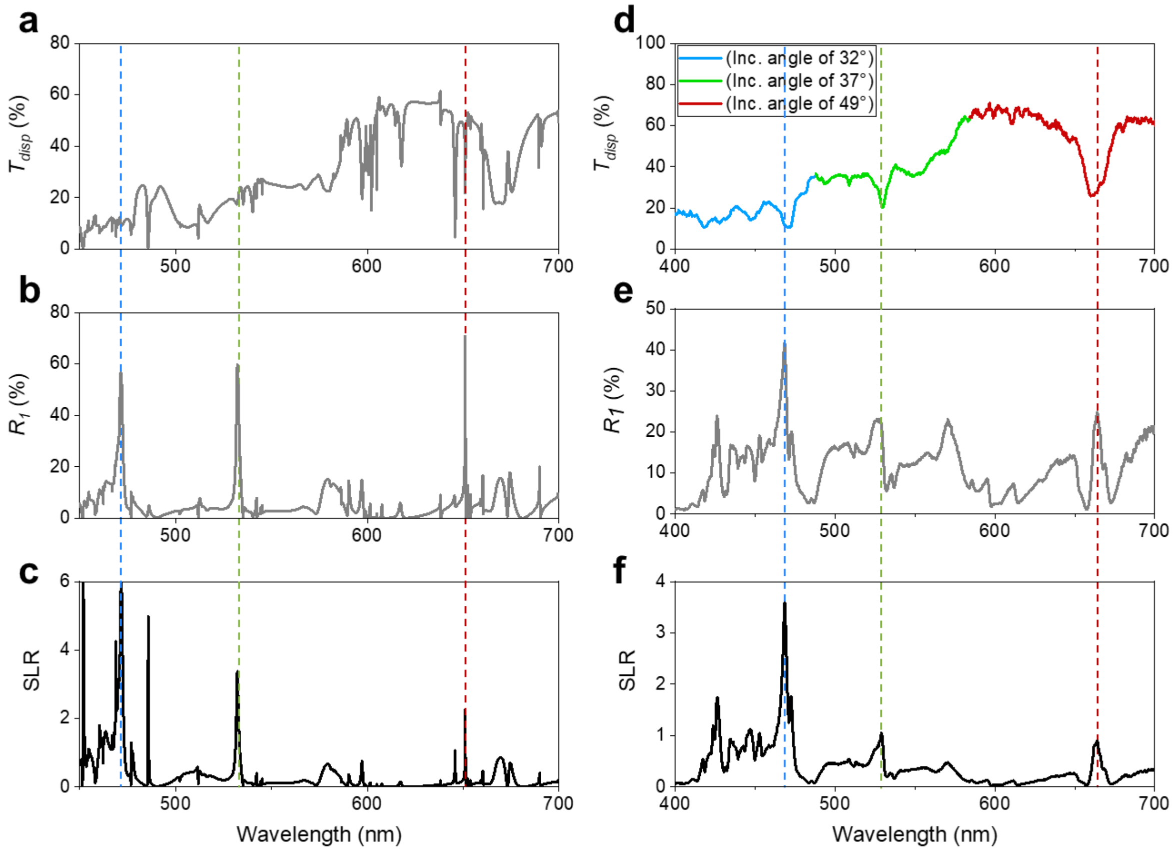

Figure S11. (a-c) Simulated results demonstrating the metasurface's ability to mitigate front projection. (a) Simulation zeroth order direct transmission T_{disp} over oblique incident, which angle obey the grating equation from first-order diffraction direction. (b) Simulation first order diffractive reflection channel R_1 (c) Numerical SLR across all three RGB design wavelengths. Several peaks except 471 nm occurred around blue channel were caused by low transmission, which is non reliable due to low R_1 value. **(d-f)** Experimental results of mitigate front projection. (d) T_{disp} measured under oblique illumination at incidence angles of 32.6º (Blue), 37.2º (Green), and 49.7º (Red), chosen to coincide with the external first-order diffraction directions of the Sample A2 at its peak resonances, respectively. The valley over visible spectrum precisely matches the first-order diffraction wavelength we measured. (e) Experimental first order diffractive reflection channel R_1. (f) Experimental SLR across visible wavelengths.

S11. FOV measurement of the AR platform

Figure S12(a) illustrates the optical configuration used to characterize the field of view (FOV) of the AR platform. A wavelength-tunable laser source (SuperK Extreme, FIU-15, NKT Photonics) equipped with a laser line tunable filter (LLTF Contrast, SR-VIS-HP8, NKT Photonics) was employed to generate monochromatic illumination at the operating wavelengths of Sample A2. The output beam was expanded by a factor of 15 using beam expanders to ensure sufficient beam diameter and spatial uniformity. A linear polarizer (PL) was used to define the polarization state of the incident light.

The expanded beam was focused onto the metasurface sample at normal incidence using Lens 1 (focal length of 50 mm). Due to the limited lateral dimension of the fabricated metasurface, this focusing step ensures efficient illumination of the active area. The diffracted beam was then collected and relayed to a camera (acA4112-20uc, Basler) using Lens 2 (focal length of 75 mm). The optical arrangement forms a configuration analogous to a 4f system, allowing direct mapping of the angular dispersion (band structure) of the metasurface onto the image plane of the camera.

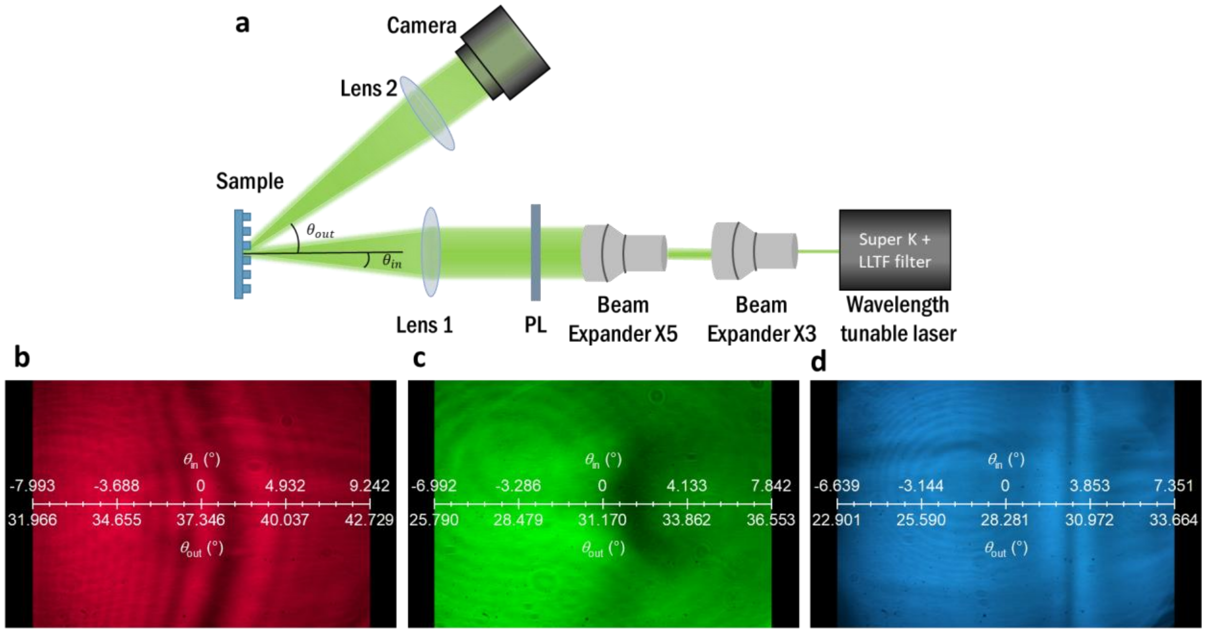

Figure S12. (a) Optical setup for FOV characterization. **(b–d)** Measured diffraction patterns at the operating wavelengths of Sample A2: 663.9 nm (b), 526.3 nm (c), and 468.2 nm (d).

Figures S12(b–d) present the measured diffraction patterns at the operating wavelengths of Sample A2 at 663.9 nm, 526.3 nm, and 468.2 nm. The measured horizontal FOV ranges from approximately 14° to 17°, depending on wavelength. The FOV in this configuration is primarily limited by the focal lengths of the relay lenses and the imaging geometry.

We note that due to the presence of resonance bands and band gaps in the angular dispersion, the diffraction intensity is not spatially uniform across the entire measured range. When uniform image quality is required—such as in AR image projection—the effective usable FOV must be selected within regions exhibiting relatively uniform and gap-free diffraction response. Under this criterion, the practically usable FOV is smaller than the maximum measured angular span reported above.

S12. Experimental setup for AR platform

Figure S13 illustrates the optical configuration used for the AR demonstration. The wavelength-tunable laser source (SuperK Extreme, FIU-15, NKT Photonics) equipped with a laser line tunable filter (LLTF Contrast, SR-VIS-HP8, NKT Photonics) was expanded by 5× using a beam expander to match the active area of the back-illuminated micro-LCD. The spectrally filtered beam first passed through a linear polarizer (PL1) before reaching the micro-LCD, where the digital image pattern was encoded via amplitude modulation. A second crossed polarizer (PL2) was placed after the micro-LCD to enhance modulation contrast and to ensure a well-defined polarization state compatible with the polarization-dependent response of the metasurface. The modulated beam was subsequently collimated by Lens 1 (focal length of 50 mm) and directed toward the metasurface sample at the external incidence angle satisfying the first-order reflective diffraction condition for the selected wavelength. In contrast to the FOV characterization setup (Supporting Information S10), which maps angular dispersion, this configuration is optimized for image projection and virtual image formation.

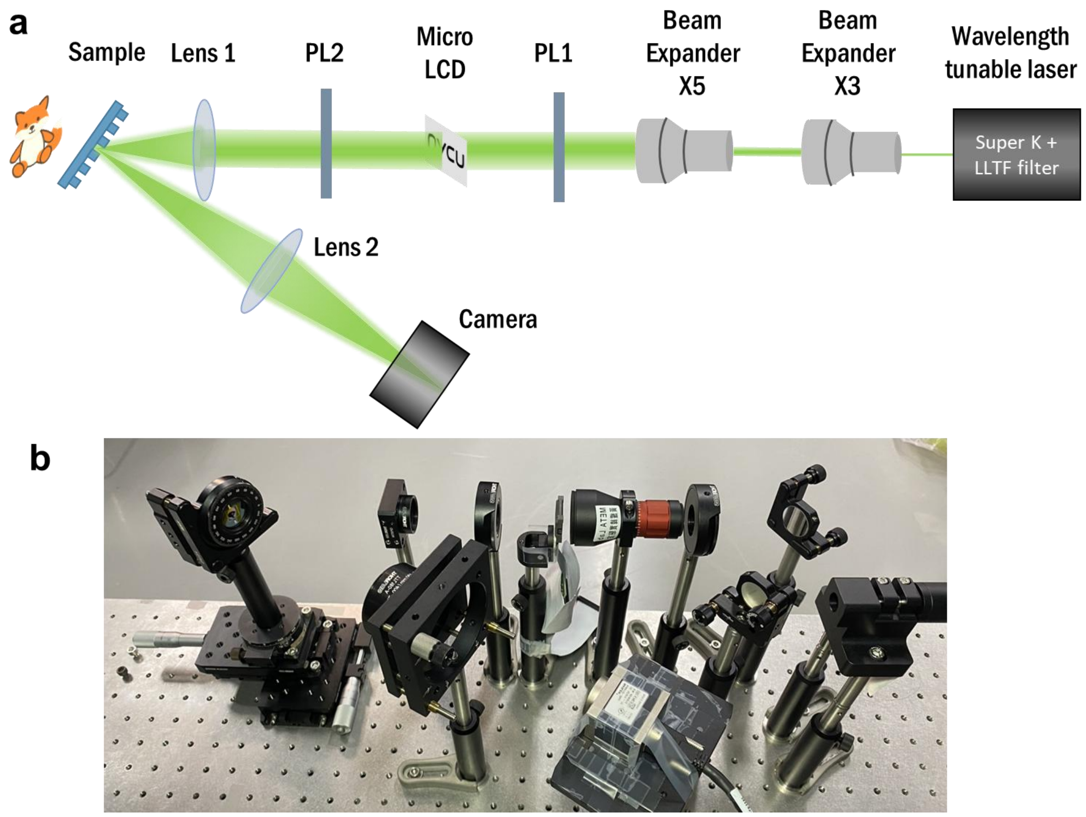

Figure S13. (a) Schematic of the AR platform experimental setup. **(b)** Photograph of the implemented optical system. The captured images were horizontally flipped to match the orientation shown in (a).

The diffracted display light was redirected normal to the sample and collected by an "eyeball model" consisting of a tube lens (TTL180-A, Thorlabs) (for better imaging quality) and

a visible-light camera (acA4112-20uc, Basler), approximating human eye perception. Ambient light from the surrounding environment transmitted through the partially transparent metasurface and was captured simultaneously by the same camera. This configuration enables superposition of the real-world scene and the laser-projected virtual content, demonstrating true see-through mixed-reality operation.

Figure S14(b–d) present the virtual images captured in the reflective configuration (R_1) using the optical setup shown in Figure S13. In this measurement, Lens 2 was replaced with a lens of focal length 100 mm to maximize the achievable horizontal FOV while avoiding mechanical interference with the incident display beam path. Under this configuration, the measured horizontal FOV is approximately 5.2° for the blue channel, 5.5° for the green channel, and 6.3° for the red channel.

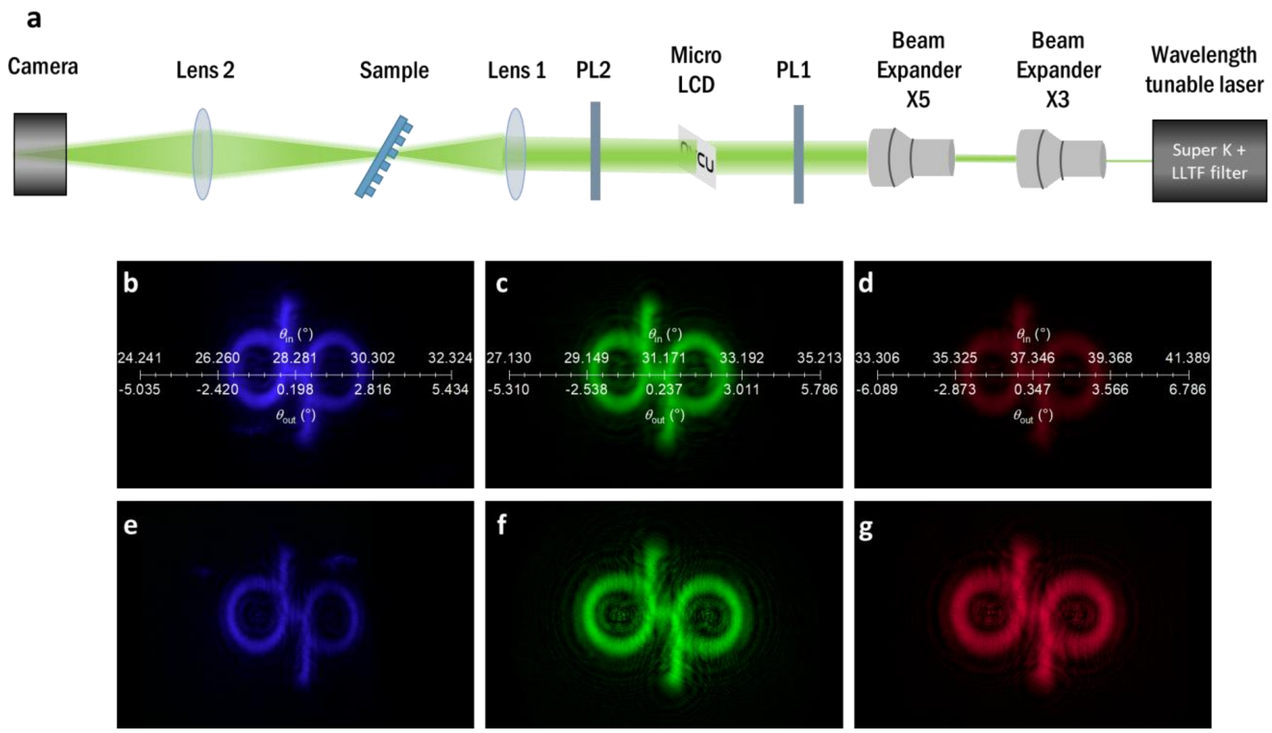

Figure S14. (a) Optical configuration for capturing the T_{disp}images. **(b–d)** Reflective-mode (R_1) images measured using the setup in Figure S13 at operating wavelengths of 663.9 nm (b), 526.3 nm (c), and 468.2 nm (d). **(e–g)** Transmitted (T_{disp}) images measured using the configuration in (a) at the corresponding wavelengths.

For comparison, the front-projection condition (T_{disp}) was also characterized using the optical arrangement illustrated in Figure S14(a). In this configuration, the image formed on the metasurface sample was transmitted through the device and imaged onto the camera using Lens 2 (focal length of 100 mm). The measured T_{disp} images for the blue, green, and red channels are shown in Figures S14(e–g), corresponding to operating wavelengths of 663.9 nm, 526.3 nm, and 468.2 nm, respectively.

Owing to the 3.59-fold enhancement in the blue channel, the R_1 image exhibits significantly higher brightness compared to the corresponding T_{disp} image. In contrast, the brightness levels of R_1 and T_{disp} images are comparable for the green and red channels. This behavior is primarily attributed to the lower experimentally realized R_1 efficiencies for these channels. Based on simulation results (Figure 4b), where SLR values of 3.37 (green) and 2.24 (red) are predicted, further improvement in fabrication fidelity is expected to enhance the reflective-mode contrast and mitigate front-projection visibility.

S13. Dispersive image compression

A pattern displayed on the micro-LCD is diffractively relayed by the combiner, its lateral size depends on the incident wavelength. Figure S15(a) demonstrated a set up to explain image compression. The micro-LCD is positioned at the focal plane of the projection lens. When an object lies exactly on axis, its rays exit the lens as a collimated beam parallel to the optical axis. However, pixels located off axis emit rays that are tilted. Consequently, identical-diameter beams originating from different LCD positions illuminate the grating at slightly different θ_i.

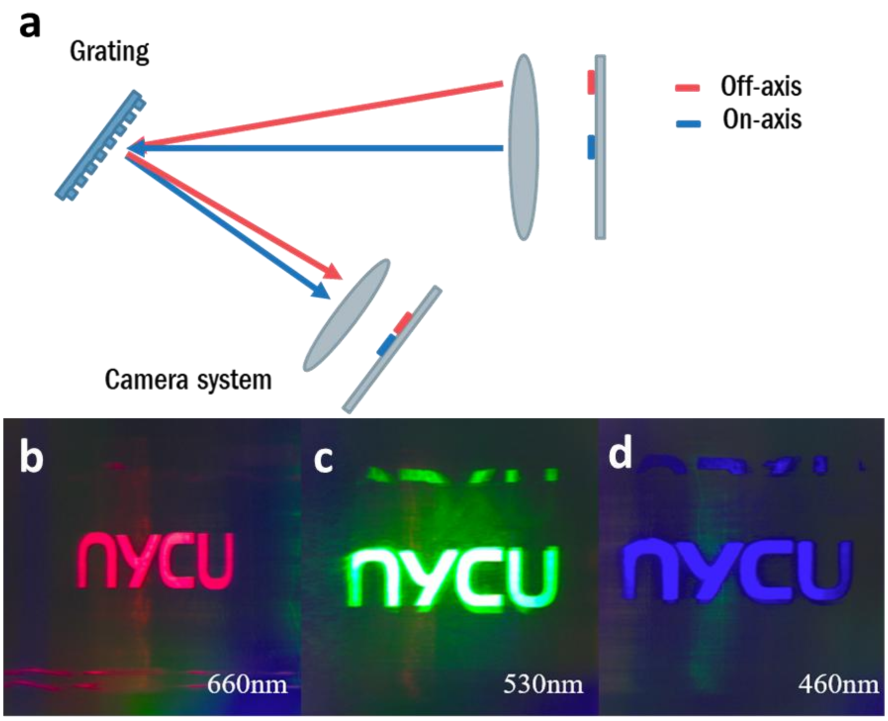

Figure S15. (a) Schmetic of image compression verifying setup. **(b-d)** The pattern displayed under red, green, and blue wavelength respectively.

For diffractive grating, the oblique incident angle of longer wavelength fulfills the grating equation in its first-order diffraction:

$$\sin\theta_i + \frac{2\pi}{\Lambda} = \sin\theta_d, \qquad \text{(S12)}$$

where θ_i is the oblique incident angle, θ_d is the corresponding diffraction angle, and Λ represents the period of the grating. Differentiate both side with θ_i we get

$$\frac{d\theta_d}{d\theta_i} = \cos\theta_i. \qquad \text{(S13)}$$

At $\theta_d \cong 0°$ when first-order diffraction light redirects normal to the grating. Equation S13 indicates the longer wavelength with larger θ_i experiences severely compression.

Because the first-order diffracted angle θ_d depends non-linearly on θ_i, the same spread in θ_i experienced different compression after diffraction. Rays that with larger θ_i were converge closer to the optical axis at the camera focal plane. This mechanism accounts for the severely reduced width of the red pattern in Figure S15(b).

A practical remedy is digital pre-compensation: once the RGB-dependent scale factors are characterised, the input frames can be resampled so that the micro-LCD can reconstruct the correct geometry. Provided that the display pixel density is sufficient, such pre-distortion introduces negligible visual artefacts. Figure S15(b-d) illustrates the same compression effect with a commercial ruled grating (GR25-1205, Thorlabs) under different operation wavelength.

S14. Simulated analysis of rainbow effect

To quantitatively evaluate potential rainbow artifacts induced by angularly dispersed ambient light, we performed numerical simulations under four distinct scenarios, as illustrated in Figure S16(a). These scenarios correspond to:

1. **Scenario 1 ($\boldsymbol{R_1}$):** Display light reflected toward the observer.

2. **Scenario 2 ($\boldsymbol{T_0}$):** Ambient light directly transmitted to the observer.

3. **Scenario 3 ($\boldsymbol{T_{-1}}$):** Ambient light diffracted into the −1 transmission order toward the observer.

4. **Scenario 4 ($\boldsymbol{T_{+1}}$):** Ambient light diffracted into the +1 transmission order toward the observer.

Because diffraction angle is wavelength-dependent, we restrict the analysis to output angles within ±10° (θ_{out}), corresponding to the angular range relevant to human eye perception in the AR configuration. The sign convention of θ_{in} and θ_{out} follows the in-plane momentum direction, with $+x$ defined as positive.

Figure S16(b) shows the corresponding incident angle (θ_{in}) ranges contributing to each scenario. Owing to the grating period of 870 nm, which is comparable to twice the shortest visible wavelength (435–700 nm range), only the ±1 transmissive diffraction orders are relevant for evaluating rainbow artifacts induced by ambient illumination.

Figure S16(c) presents the simulated efficiency colormaps as functions of wavelength and output angle θ_{out} for the four scenarios. Scenario 1 (display reflection) exhibits multiple localized high-efficiency regions, consistent with the guided-mode resonance features analyzed in Supporting Information S4. Scenario 2 (direct ambient transmission) shows a broadly complementary trend relative to Scenario 1, with comparatively higher transmission efficiency in the red spectral region. Scenarios 3 and 4 correspond to ambient light entering the eye via −1 and +1 transmissive diffraction orders, respectively. The −1 order (Scenario 3) exhibits overall low efficiency across the visible spectrum and is therefore negligible. The +1 order (Scenario 4) shows moderate efficiency, particularly in the green wavelength range (approximately 500–514 nm), where the diffracted intensity can locally exceed that of direct transmission (Scenario 2). This spectral region represents the primary contributor to potential rainbow artifacts.

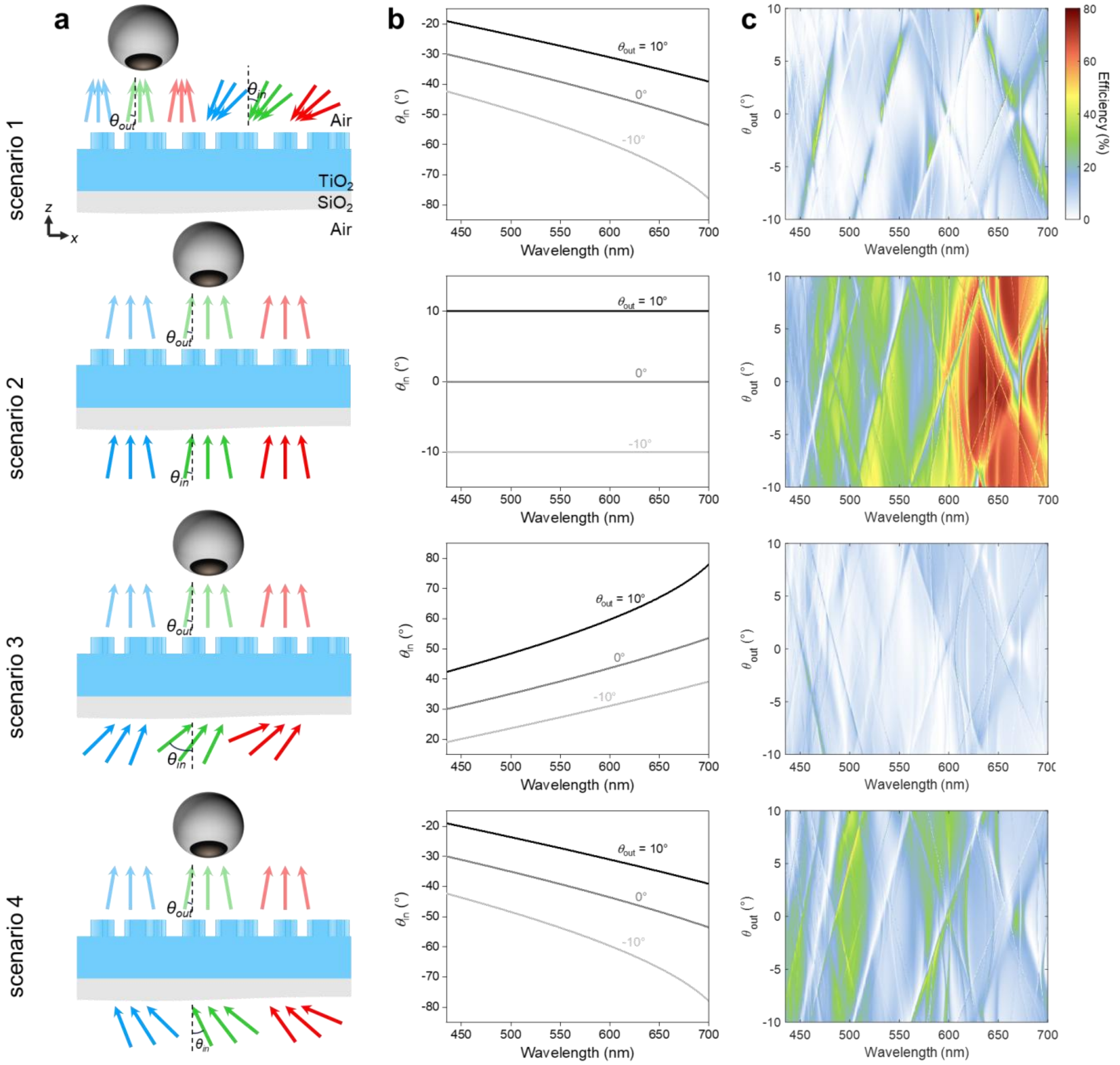

Figure S16. (a) Schematic illustration of the four scenarios considered in the rainbow-effect analysis, corresponding to R_1, T_0, T_{-1}, and T_{+1}. Output angles within ±10° are considered. **(b)** Corresponding incident angle ranges (θ_{in}) as a function of wavelength for each scenario. **(c)** Simulated efficiency colormaps as functions of wavelength and output angle θ_{out} for the four scenarios.

Figure S17 summarizes the angularly averaged efficiencies within ±10° of θ_{out}. For Scenario 1 (display reflection), the averaged peak efficiencies for the blue, green, and red channels are 9.24%, 14.0%, and 16.7%, respectively. Enhancement of virtual image brightness can be achieved by increasing display power within these bands. For ambient light, the averaged direct transmission efficiency (Scenario 2) is approximately 27% in the blue and green regions and 53% in the red region. The averaged efficiency of the −1 transmission order (Scenario 3) is approximately 5% and can be considered negligible. In contrast, the +1 transmission order

(Scenario 4) exhibits locally elevated efficiency in the green spectral range, indicating that this wavelength region warrants particular attention in rainbow-effect mitigation.

To further suppress potential rainbow artifacts, two complementary strategies may be adopted. At the device-design level, the transmissive diffraction efficiency can be incorporated into the topology-optimization figure of merit to penalize undesired angular dispersion of ambient light. At the system level, angular filtering components—such as micro-louver–based privacy layers—can reduce large-angle ambient illumination before it interacts with the metasurface. These approaches provide practical pathways for minimizing rainbow artifacts in future implementations.

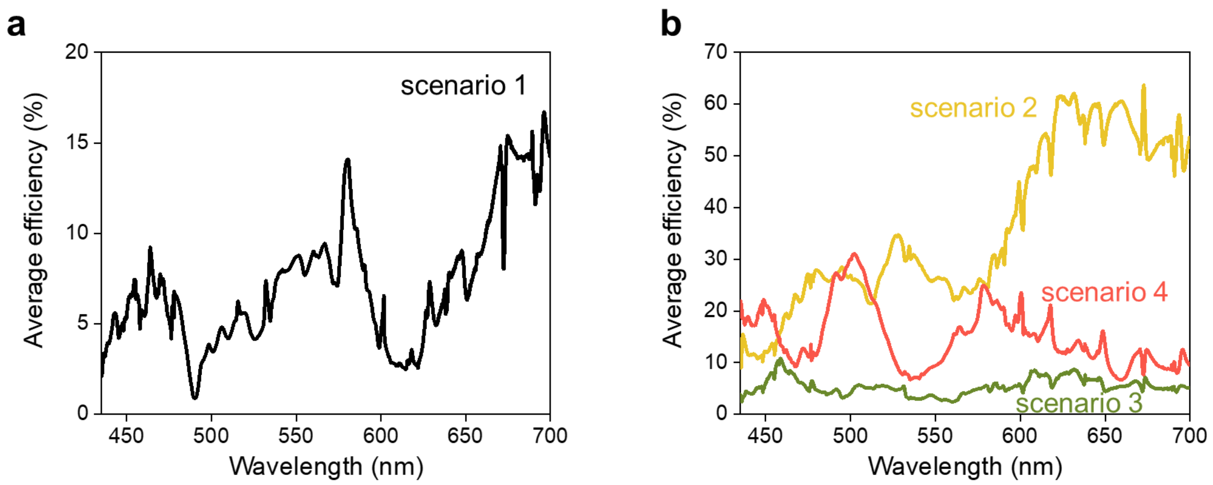

Figure S17. (a) Angularly averaged R_1 efficiency within ±10° of θ_{out} (Scenario 1). **(b)** Angularly averaged transmission efficiencies within ±10° of θ_{out} for Scenarios 2–4.
